# Alleviation of liver cirrhosis and associated portal-hypertension by *Astragalus* species in relation to their UPLC-MS/MS metabolic profiles: a mechanistic study

**DOI:** 10.1038/s41598-022-15958-1

**Published:** 2022-07-13

**Authors:** Reham S. Ibrahim, Nesrine S. El-Mezayen, Alaa A. El‐Banna

**Affiliations:** 1 Department of Pharmacognosy, Faculty of Pharmacy, Alexandria University, Alexandria, 21521 Egypt; 2 Department of Pharmacology, Faculty of Pharmacy, Pharos University in Alexandria, Alexandria, Egypt

**Keywords:** Biochemistry, Chemical biology, Plant sciences, Gastroenterology

## Abstract

Liver cirrhosis is a late-stage liver disease characterized by excessive fibrous deposition triggering portal-hypertension (PH); the prime restrainer for cirrhosis-related complications. Remedies that can dually oppose hepatic fibrosis and lower PH, may prevent progression into decompensated-cirrhosis. Different *Astragalus*-species members have shown antifibrotic and diuretic actions with possible subsequent PH reduction. However, *A.spinosus and A.trigonus* were poorly tested for eliciting these actions. Herein, *A.spinosus* and *A.trigonus* roots and aerial parts extracts were subjected to comprehensive metabolic-fingerprinting using UHPLC-MS/MS resulting in 56 identified phytoconstituents, followed by chemometric untargeted analysis that revealed variable metabolic profiles exemplified by different species and organ types. Consequently, tested extracts were *in-vivo* evaluated for potential antifibrotic/anticirrhotic activity by assessing specific markers. The mechanistic prospective to induce diuresis was investigated by analyzing plasma aldosterone and renal-transporters gene-expression. Serum apelin and dimethylarginine-dimethylaminohydrolase-1 were measured to indicate the overall effect on PH. All extracts amended cirrhosis and PH to varying extents and induced diuresis via different mechanisms. Further, An OPLS model was built to generate a comprehensive metabolic-profiling of *A.spinosus* and *A.trigonus* secondary-metabolites providing a chemical-based evidence for their efficacious consistency. In conclusion, *A.spinosus* and *A.trigonus* organs comprised myriad pharmacologically-active constituents that act synergistically to ameliorate cirrhosis and associated PH.

## Introduction

Liver cirrhosis is the end-stage of all chronic liver diseases that often brings about liver cancer and premature death^1^. Chronic liver diseases encompass liver injury that proceeds to hepatic fibrosis then cirrhosis with subsequent fibrotic distortion of hepatic microvasculature triggering portal hypertension (PH)^2^. Transdifferentiation of quiescent, vitamin-A-storing hepatic stellate cells (HSCs) into proliferative, fibrogenic myofibroblasts is the central driver of fibrosis progression^3^. This transdifferentiation is induced by cytokines and proteomic mediators including; transforming growth factor-β (TGF-β), a key profibrogenic cytokine and a central regulator of liver disease progression^4^, and retinoid X receptors (RXRs), a modulator of cell proliferation and the expression of profibrotic genes that play a major role in HSC activation^5^. Subsequently, massive accumulation of extracellular matrix (ECM) occurs with aberrant regulation of its turnover via tissue inhibitor metalloproteinases and matrix metalloproteinases (MMPs). Specifically, MMP–2 (Gelatinase A) expression was found to have high fibrosis/cirrhosis diagnostic accuracy with many fold increase in fibrotic/cirrhotic liver compared to normal one^6^.

Cirrhosis progression encloses two main phases; the first is a relatively slow, silent and asymptomatic phase that is referred to as “compensated cirrhosis”. The second phase is the “decompensated cirrhosis” phase that manifests serious complications; such as ascites, hemorrhage, jaundice, or hepatic encephalopathy. All these clinical phenotypes are derived by PH and are accompanied with rapid deterioration towards death or liver transplantation^7,8^. Therefore, the estimation of both intrahepatic and systemic hemodynamic aspects is strongly recommended for cirrhotic patients, for instance by assessing serum apelin or dimethylarginine dimethylaminohydrolase-1 (DDAH-1) that metabolizes the asymmetric dimethylarginine (ADMA), the competitive endogenous inhibitor of endothelial nitric oxide synthase (eNOS) that synthetizes nitric oxide (NO)^9,10^. In cirrhosis, hepatic NO levels control intrahepatic vascular tone with associated elevated sinusoidal vascular resistance^10^.

Since advanced fibrosis and PH are the strongest predictors of decompensated cirrhosis, their regression via suitable therapeutic approach is expected to be associated with lower rates of decompensation and death. Currently, there are no approved therapies that can ameliorate fibrosis^11^, but, the use of diuretics, is indispensable for PH and edema control in cirrhotic patients^12,13^. Diuretics can affect diverse transporters that exist at different sites of the nephron. Different nephron areas differ in the percentage of the reabsorbed sodium; the proximal tubule; accounts for the majority of sodium reabsorption, the thick ascending limb of the loop of Henle reabsorbs a total of 20–25% of the filtered sodium, the distal convoluted tubule where the reabsorption of 5–10% of glomerular filtrate occurs and the collecting ducts where less than 3% of the filtrate is reabsorbed^14^. In addition, at the apical and basolateral membranes of kidney proximal tubule, aquaporin-1 facilitates water reabsorption that causes urine concentration^15^. Thus, identifying the exact site of action of a compound with potential diuretic effect is crucial for predicting the extent of its diuretic effect.

*Astragalus* genus is the most prevalent in the Fabaceae family containing more than 2,500 species. A broad range of pharmacological compounds have been formerly isolated from different *Astragalus* species roots and aerial parts indicating pronounced pharmacological potential of this genus^16^. Plants from this genus have been widely used as an alternative medicine in many countries for treating chronic liver diseases. The most extensively studied species for its influence in protecting from or ameliorating chronic liver diseases and fibrosis is *A. membranaceus*^17–19^. Other *Astragalus* species that were proved to have hepatoprotective activity are *A. complanatus* herb and root^20^, *A. gummifer* flavonoids^21^ and *A. kahiricus* root extract^22^. In addition, different *Astragalus* species had shown powerful diuretic effect including *Astragalus membranaceus*, *Astragalus glycyphyllos* and *Radix Astragali*^23^. On the other hand, the influence of *A. spinosus* and *A. trigonus* on hepatic fibrosis/cirrhosis and associated PH is poorly studied.

From a chemical perspective, numerous bioactive phytoconstituents were identified in *Astragalus* species and they can be classified into flavonoids, phenolic acids, terpenoids and polysaccharides^24^. Amongst which, flavonoids and terpenoids were extensively studied in Egyptian *Astragalus* species^25–29^. However, aforementioned studies were solely focusing on particular chemical markers; chiefly astragalosides and flavonoids rather than comprehensive profiling of the entire metabolome.

Recently, liquid chromatography-mass spectrometry (LC–MS) has been broadly employed in metabolic fingerprinting owing to its wide range of separation, high selectivity, and sensitivity^30^. In addition, tandem MS generates informative fragment peaks, forming fingerprints specific to the detected molecules^31^. Multivariate data analysis can subsequently correlate the attained holistic metabolome with the implemented therapeutic effects aiming to discovery of potential health relevant phytoconstituents from complex chemical matrices such as plant extracts even with no prior isolation and hence shortens the way toward new lead discovery^32^.

Herein, two different Egyptian *Astragalus* species, namely, *A. spinosus* and *A. trigonus* roots and aerial parts extracts were assessed *in-vivo* for hepatoprotective potential against liver cirrhosis. The aim was to identify the more prominent diuretic mechanism exerted by each extract and consequent reduction of PH in rats. Further, these extracts were subjected to UHPLC-MS/MS analysis to explore the variability of the metabolic profiles exemplified by different species and organ types. An Orthogonal Projection to Latent Structures (OPLS) model was built in order to correlate the metabolite profiles with therapeutic activity to explore putative biomarkers that prospectively alleviate liver cirrhosis and associated PH. The current study represents the first comprehensive metabolic profiling of *A. spinosus* and *A. trigonus* roots and aerial parts secondary metabolites that can represent a chemical-based evidence for their efficacious consistency.

## Results

### *Astragalus* species and organ types metabolites profiling via UPLC-MS/MS analysis

**Figure 1:**
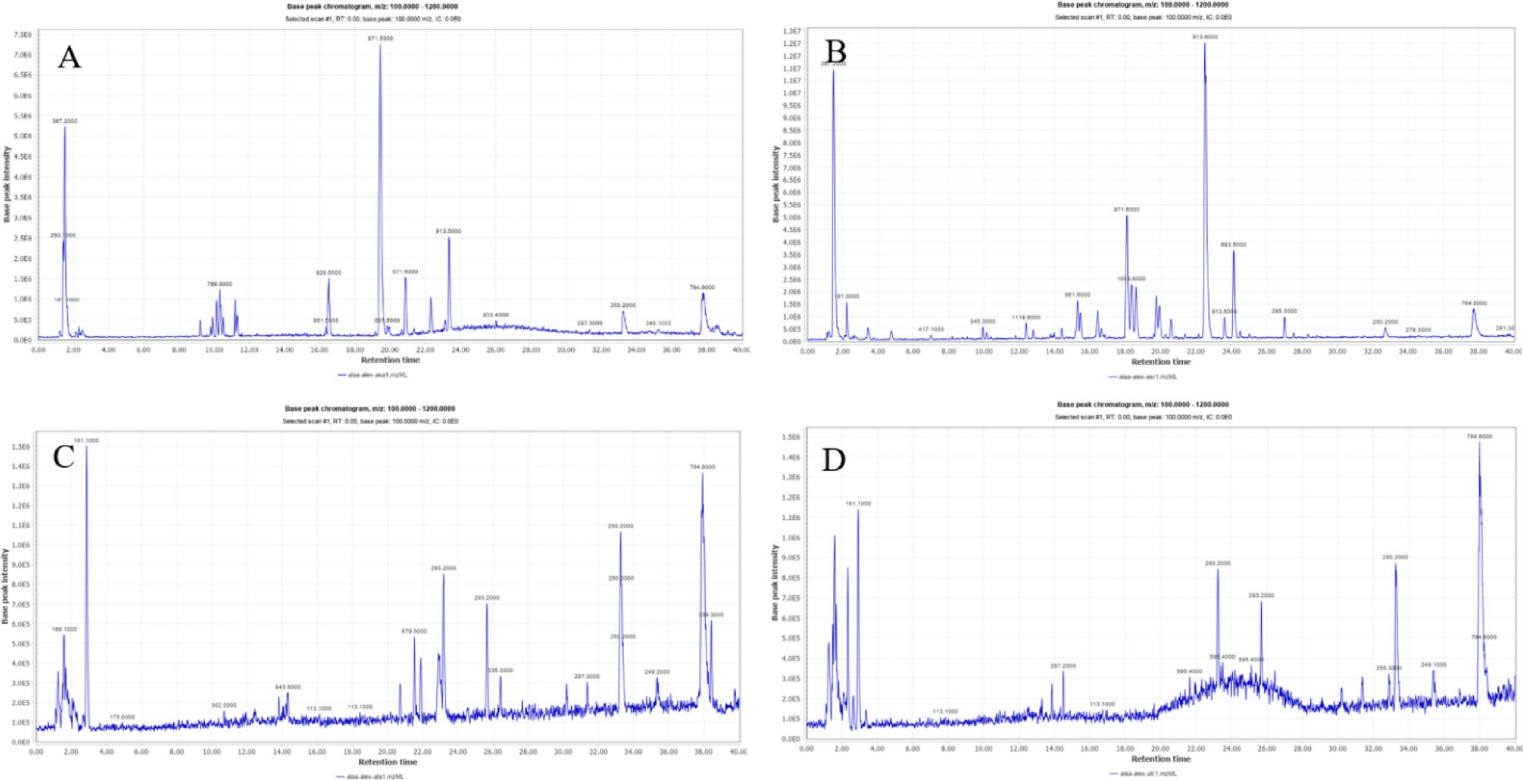
UPLC-ESI- TQD-MS base peak chromatograms of *Astragalus* methanolic extracts in negative ionization mode. *A. spinosus* aerial parts (**A**), *A. spinosus* roots (**B**), *A. trigonus* aerial parts (**C**) and *A. trigonus* roots (**D**) chromatograms.

The secondary metabolome heterogeneity among *A. spinosus* and *A. trigonus* roots and aerial parts was assessed using untargeted UPLC-MS metabolite profiling coupled with multivariate data analyses for the first time. The base peak chromatograms of the four extracts are represented in Fig. 1. A total of 146 chromatographic peaks were observed (Table S1) of which 56 belong to different classes were identified viz., phenolic acids, flavonoids, saponins, and fatty acids as listed in Table 1. Further, all metabolites identified in *Astragalus* samples were relatively quantified by using the calibration curves as described under Supplementary Material Table S2.

### Unsupervised pattern recognition analysis of *Astragalus* species and organs metabolic profiles

Unsupervised data analysis clustering techniques i.e., principal component analysis (PCA) and hierarchical clustering analysis (HCA) were implemented for determining metabolites heterogeneity among *Astragalus* species and organs. These chemometric tools are progressively applied for chemotaxonomic classification of plant species^33^.

**Figure 2:**
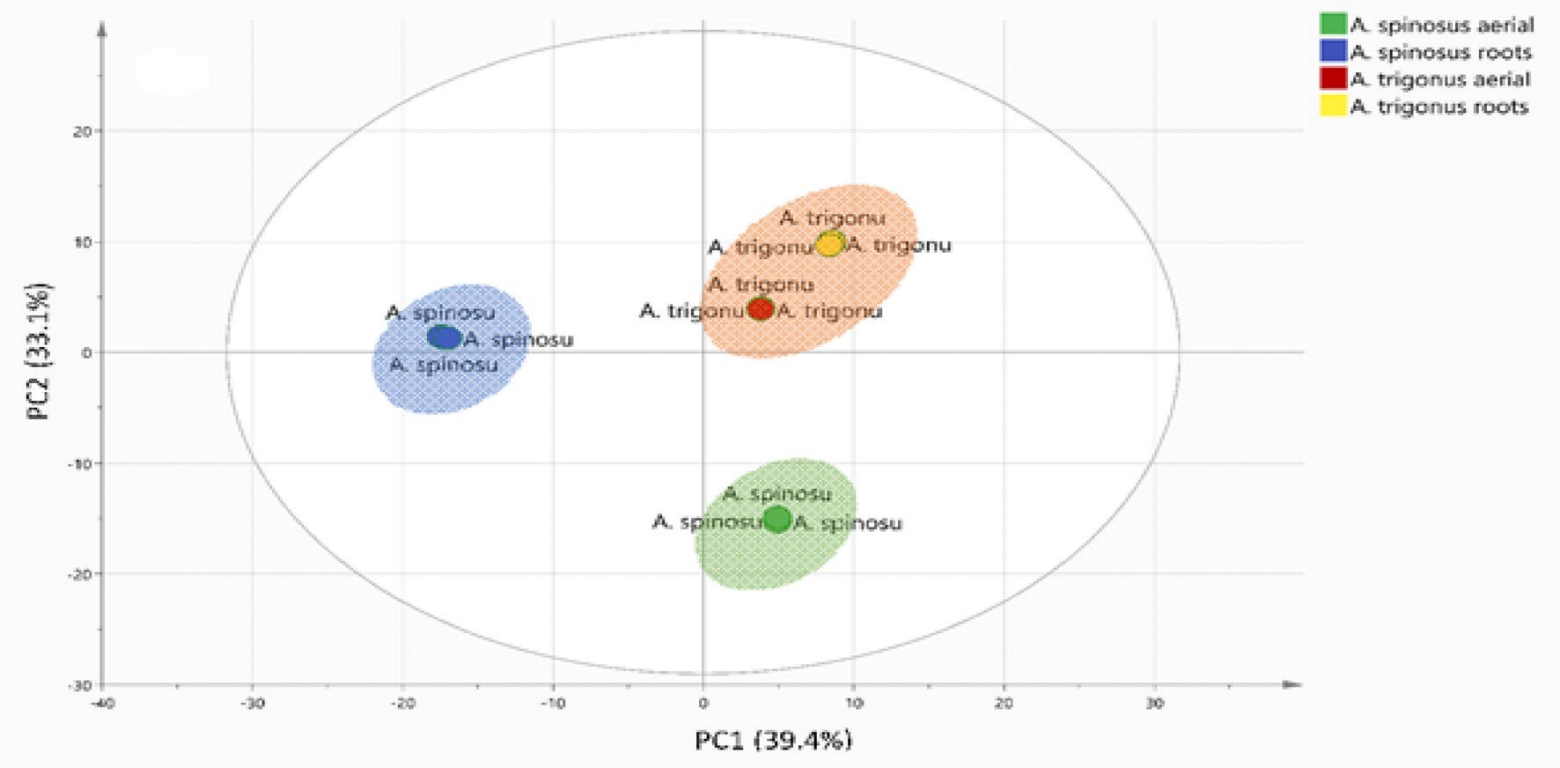
Score scatter plot of principal component analysis using UPLC-ESI- TQD-MS, n = 3. represented by first two components.

A PCA model was constructed using the UPLC-ESI- TQD -MS extracted mass intensities (Table S1). The retrieved scatter plot was prescribed using first two principal components (PC1 and PC2) accounting for 72.5% of the total variance (Fig. 2). Generally, all replicates from each species were clustered together and separated from other species indicating the reproducibility of the applied analytical method. *A. trigonus* aerial and root samples were positioned on the positive sides of both PC1 and PC2, whereas *A. spinosus* root samples were placed on the negative side of PC1 and positive side of PC2. Finally, *A. spinosus* aerial samples were individually clustered at the lower right quartile of the score plot.

**Figure 3:**
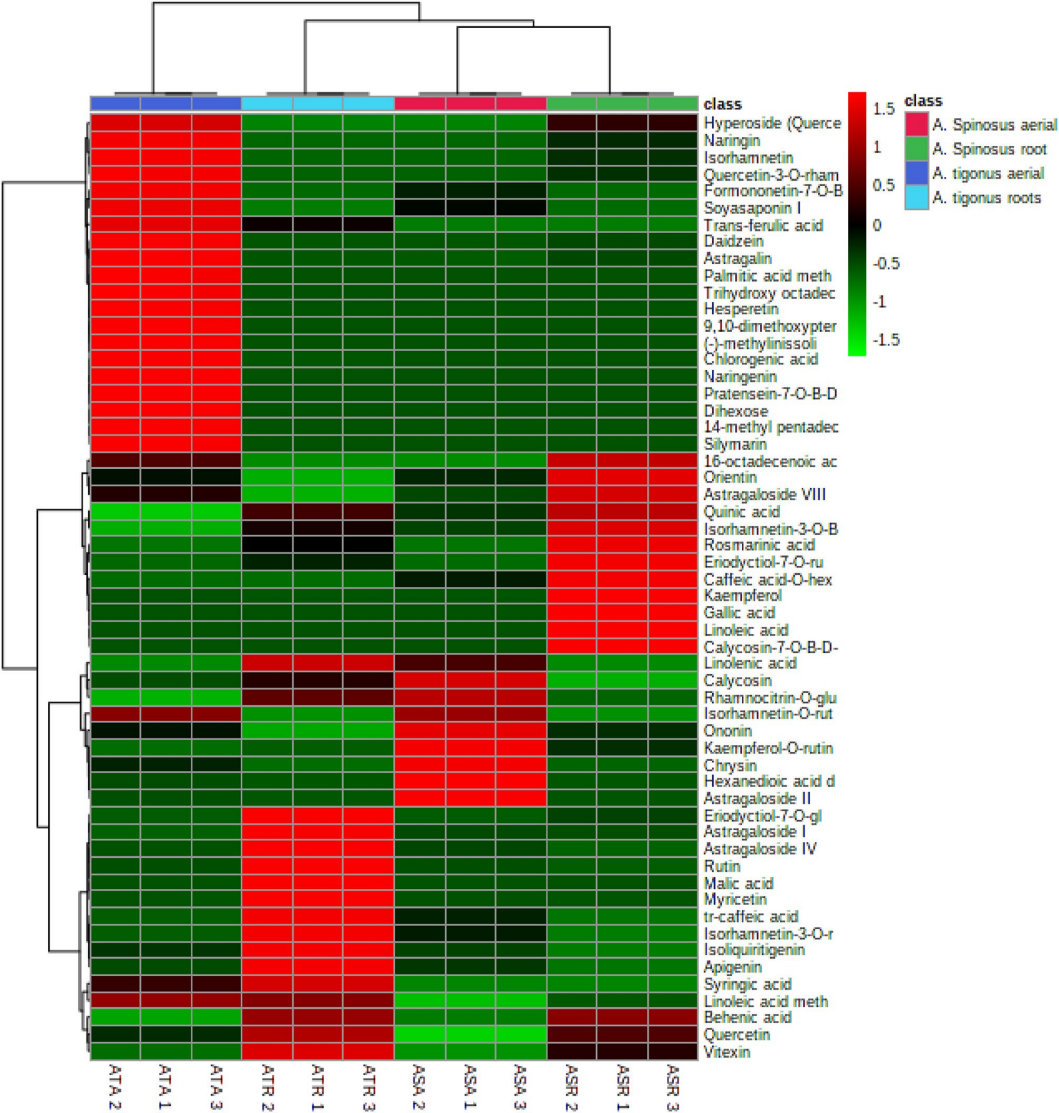
HCA- heat map showing the most discriminatory metabolites in each cluster.

HCA-heat map was further constructed using compounds’ peak intensities showing a dendrogram in which three clusters were designated (Fig. 3). An examination of clustering pattern disclosed that samples of *A. spinosus* aerial parts formed the first cluster. Whereas the second cluster comprised of *A. spinosus* root samples*. A*. *trigonus* aerial and root subclusters constituted together the third cluster. Further analysis of dendrogram revealed the most discriminatory metabolites likely to be of chemotaxonomic significance in *A. spinosus* and *A. trigonus* as well as organ-type distinction (Fig. 3).

### Effect of Different *A. spinosus* and *A. trigonus* Extracts on Liver Cirrhosis Markers

#### *In-vivo* influence and OPLS analysis of different extracts on TGF-β

**Figure 4:**
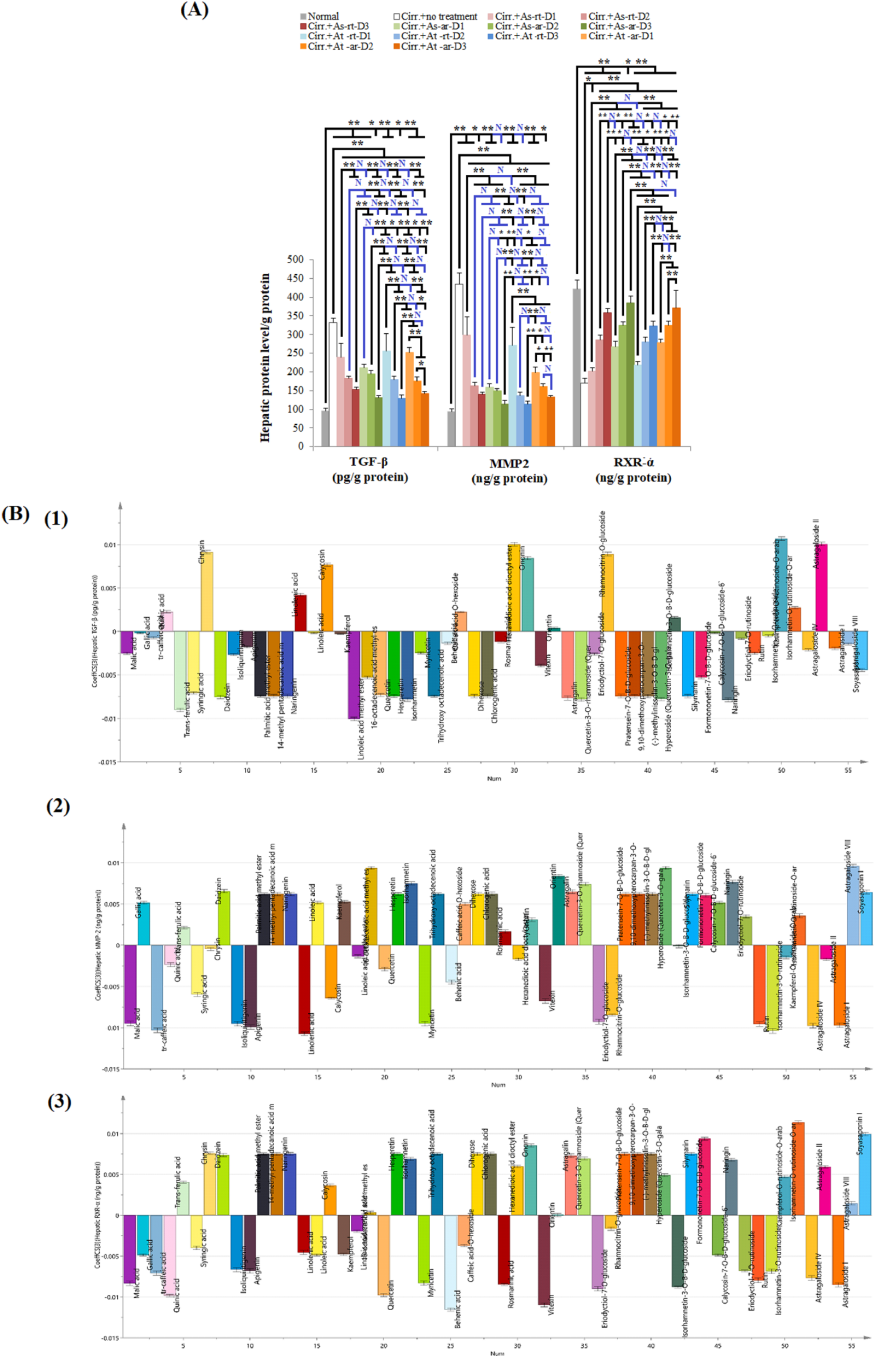
The effect of different *Astragalus* extracts on liver cirrhosis markers and their coefficient plot of OPLS model. (**A**): Comparison between different groups according to hepatic concentration of different cirrhosis parameters, (**B**): Coefficient plot of OPLS model for liver cirrhosis parameters; (1) TGF-ꞵ (2) MMP-2 (3) RXR. ANOVA test was used to compare between the different groups with Post Hoc Test (Tukey) to compare different groups. *: Statistically significant at *p* ≤ 0.05, **: Statistically significant at *p* ≤ 0.001, N: Statistically non-significant (*p* > 0.05), n = 8; all results are presented as mean ± SD. (Cirr.: induced cirrhosis by i.p. administration of CCl_4_ (diluted 1:6 with mineral oil) as follows: The first 10 doses were received every 5 days, the subsequent 10 doses were administered every 4 days, and the last 7 doses were given every 3 days, As: *Astragalus spinosus*, At: *Astragalus trigonus*, ar: aerial organs, rt: root organs, D1: low dose, D2: medium dose, D3: high dose, MMP2: matrix metalloproteinase 2, TGF-ꞵ: transforming growth factor beta, RXR: retinoid-X receptor, ALT: Alanine aminotransferase and AST: aspartate aminotransferase.

In the present work, significant elevation in TGF-β levels were observed in untreated rats’ livers compared to normal negative control rats (*p* < 0.001). All tested doses of *A. spinosus* and *A. trigonus* aerial and root samples extracts significantly reduced hepatic TGF-β levels (*p* < 0.001). The most potent reduction in hepatic TGF-β levels occurred upon the administration of *A. spinosus* aerial part *(*As-ar) extract even upon administration of a low dose; as the low dose showed the least TGF-β levels as compared to the low dose of all other tested extracts (*p* < 0.001). The least TGF-β levels were observed in As-ar and *A. trigonus* root extract (At-rt). However, hepatic TGF-β levels in rats treated with high dose As-ar were not statistically significant compared to those in rats treated with the high doses At-rt or *A. trigonus* aerial parts (At-ar) extracts (p values are 1.00 and 0.992, respectively), Fig. 4A.

Conducting OPLS model revealed that the top efficient compounds modulating hepatic TGF-β and reducing its level were linoleic acid methyl ester, ferulic acid and naringin (naringenin 7-O-neohesperidoside), Figs. 4b1 and S1A. The former compound existed in the four tested extracts, while ferulic acid was detected only in the extracts of the At-ar and At-rt, and naringin was found in As-rt and At-ar, supplementary Tables S1 and S3.

#### *In-vivo* influence and OPLS analysis of different extracts on MMP-2

In the present investigation, there was 4.6 fold increase in hepatic MMP-2 concentration in untreated cirrhotic livers compared to normal livers (*p* < 0.001). Effect of different extracts on hepatic MMP-2 was very similar to their effect on TGF-β; all treatments resulted in significant reduction in hepatic MMP-2 levels compared to the untreated positive control group (*p* < 0.001). Also, As-ar revealed the greatest potency in hepatic MMP-2 reduction; MMP-2 level in As-ar-low dose-treated group was comparable to its level in As-rt and At-ar high dose-treated groups (*p* = 0.828 and 0.319, respectively) and At-rt medium dose-treated group (*p* = 0.672), Fig. 4A. Moreover, similar to TGF-β results, MMP-2 levels in As-ar and At-rt high dose-treated groups were the least inspite of being not statistically significantly different from other groups treated with high doses As-rt or At-ar.

According to the OPLS model, the phytoconstituents that were found to be highly correlated to hepatic MMP-2 levels modulation were linolenic acid, tr-caffeic acid and isorhamnetin-3-O-rutinoside, Figs. 4B2 and S1A. In the tested *Astragalus* species, linolenic acid was detected in both As-ar and At-rt. Tr-Caffeic acid was identified in all tested extracts except for As-rt, whereas, isorhamnetin-3-O-rutinoside was found in the four tested extracts, supplementary Tables S1 and S3.

#### *In-vivo* influence and OPLS analysis of different extracts on RXR

In the present study, RXR-α mean level in normal livers was about 2.5 folds greater than that in cirrhotic livers. Administration of different extracts significantly restored RXR-α levels compared to untreated rats with liver cirrhosis. Upon comparing extracts potency in restoring RXR-α, that is the ability to exert the most significant restoration with the administration of the low dose of the extract, extracts of the aerial parts of *A. spinosus* and *A. trigonus* showed the greatest potency with no statistically significant difference between them (*p* = 0.981). The maximum restoration capacity of RXR-α level was observed with the high dose As-ar. The order of extracts efficacy in restoring RXR-α level was As-ar, At-ar, As-rt then At-rt in descending order, Fig. 4A.

As revealed by OPLS model, activity on RXR-α is mainly attributed by isorhamnetin-O-rutinoside-O-arabinoside, soyasaponin I and formononetin-7-O-glucoside-6``-O-malonate, Figs. 4b3 and S1A. Isorhamnetin-O-rutinoside-O-arabinoside was only detected in the aerial parts of both *A. spinosus* and *A. trigonus,* and was not detected in their roots. Soyasaponin I was found in all tested extracts except for At-rt, while formononetin-7-O-glucoside-6``-O-malonate was detected in aerial parts and roots of both tested species*,* supplementary Tables S1 and S3.

#### *In-vivo* influence and OPLS analysis of different extracts on hepatocytes integrity and liver synthetic function

**Figure 5:**
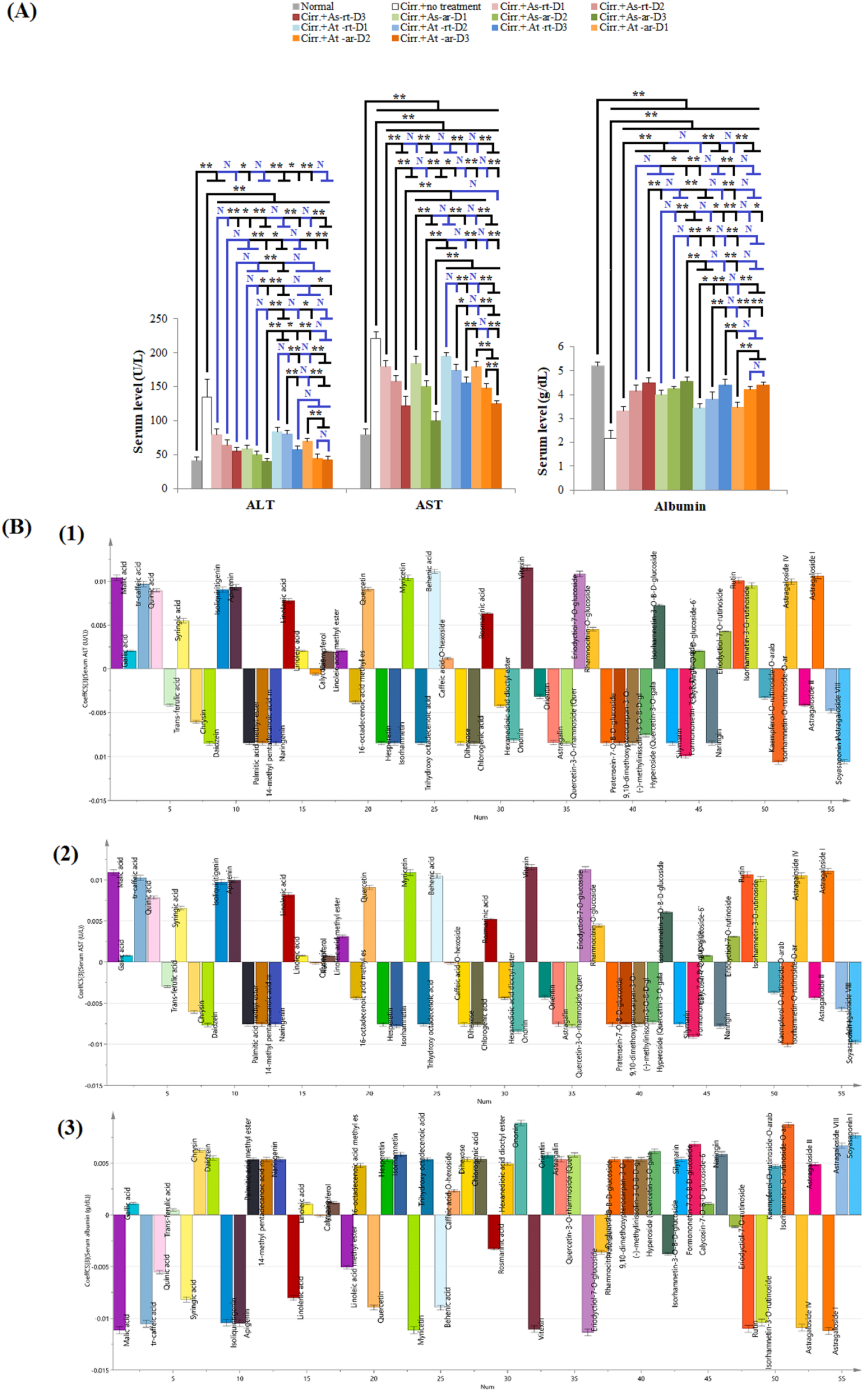
The effect of different *Astragalus* extracts on serum markers of hepatocytes integrity and liver synthetic function and their coefficient plot of OPLS model. (**A**): Comparison between different groups according to serum markers of hepatocytes integrity and liver synthetic function, (**B**): Coefficient plot of OPLS model for serum markers of hepatocytes integrity and liver synthetic function; (1) ALT (2) AST (3) albumin. ANOVA test was used to compare between the different groups with Post Hoc Test (Tukey) to compare different groups. *: Statistically significant at p ≤ 0.05, **: Statistically significant at *p* ≤ 0.001, N: Statistically non-significant (*p* > 0.05), n = 8; all results are presented as mean ± SD. (Cirr.: induced cirrhosis by i.p. administration of CCl_4_ (diluted 1:6 with mineral oil) as follows: The first 10 doses were received every 5 days, the subsequent 10 doses were administered every 4 days, and the last 7 doses were given every 3 days, As: *Astragalus spinosus*, At: *Astragalus trigonus*, ar: aerial organs, rt: root organs, D1: low dose, D2: medium dose, D3: high dose, ALT: Alanine aminotransferase and AST: aspartate aminotransferase.

Induction of cirrhosis resulted in 3.3 and 2.8 folds increase in Alanine aminotransferase (ALT) and aspartate aminotransferase (AST) serum levels, respectively and 2.4 fold decrease in serum albumin level, Fig. 5A. The high and/or intermediate doses of three of the tested *Astragalus* extracts (As-rt D3, As-ar D2, As-ar D3, At-ar D2 and At-ar D3) were able to return ALT to its basal levels. P-values for comparing these extracts to normal were 0.109, 0.694, 1.00, 1.00 and 1.00, respectively. On the other hand, though no extract was able to return AST or albumin to normal base value, they all succeeded to significantly reduce AST and increase albumin level compared to positive control untreated rats (p values for comparing to normal rats or positive controls were < 0.001). Of notice, At-ar D3 showed almost similar action on ALT and albumin as As-ar D3 with no statistically significant difference between them. Nevertheless, no other group showed similar action on AST as As-ar D3 that revealed significantly less AST levels compared to all other treated groups (*p* < 0.001), Fig. 5A.

Top detected compounds using OPLS model that scored activities against AST and ALT were isorhamnetin-O-rutinoside-O-arabinoside, soyasaponin I and formononetin-7-O-glucoside-6``-O-malonate in descending order. Whilst the top scoring compounds against albumin was ononin (Formononetin-7-O-glucoside), followed by isorhamnetin-O-rutinoside-O-arabinoside and soyasaponin I, Figs. 5B and S1B. Noticeably, all these compounds were detected in the arial parts of the tested *Astragalus* species*,* supplementary Tables S1 and S3.

### Prediction of the Diuresis Mechanism of Different *Astragalus* Extracts

Different *Astragalus* species had shown powerful diuretic effect including *Astragalus membranaceus*, *Astragalus glycyphyllos* and *Radix Astragali*^23^. Limited data pointed to the diuretic effect of the species that we are concerned about in the present study. Thus, a screening of the potential diuretic effect of the four examined extracts and thorough investigation of the exact mechanism implying their diuretic action were currently performed.

#### Extracts acting at the proximal convoluted tubule:

**Figure 6:**
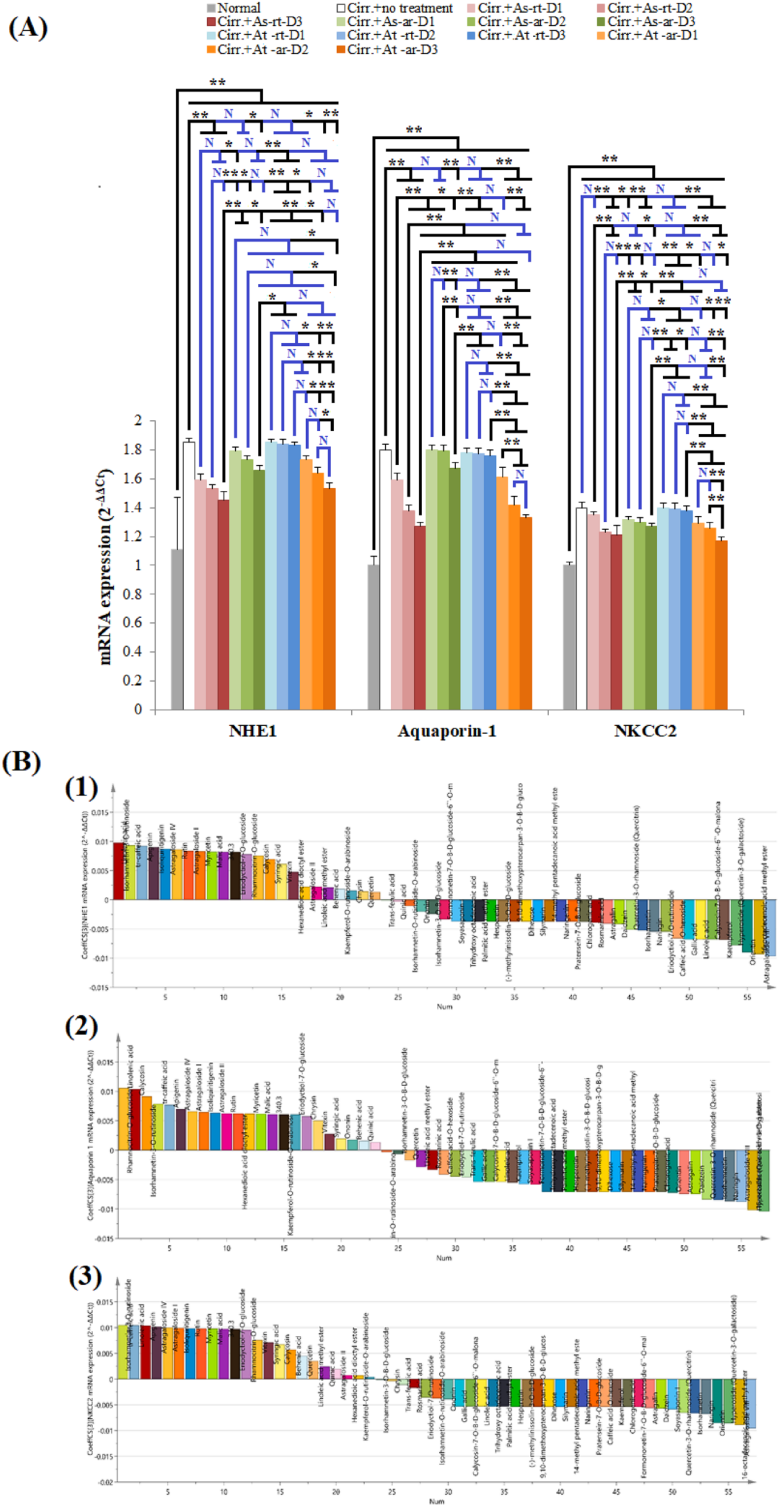
The effect of different *Astragalus* extracts on mRNA expression of proximal convoluted tubule and the thick ascending limb of the loop of Henle transporters and their coefficient plot of OPLS model. (**A**): Comparison between different groups according to mRNA expression of different renal transporters acting on proximal convoluted tubule and loope of Henle, (**B**): Coefficient plot of OPLS model for diuretic mechanisms; (1) NHE-1 (2) Aquaporin (3) NKCC2. ANOVA test was used to compare between the different groups with Post Hoc Test (Tukey) to compare different groups. *: Statistically significant at *p* ≤ 0.05, **: Statistically significant at p ≤ 0.001, N: Statistically non-significant (*p* > 0.05), n = 8; all results are presented as mean ± SD. (Cirr.: induced cirrhosis by i.p. administration of CCl_4_ (diluted 1:6 with mineral oil) as follows: The first 10 doses were received every 5 days, the subsequent 10 doses were administered every 4 days, and the last 7 doses were given every 3 days, As: *Astragalus spinosus*, At: *Astragalus trigonus*, ar: aerial organs, rt: root organs, D1: low dose, D2: medium dose, D3: high dose, NHE1: sodium–hydrogen antiporter 1, NKCC 2: Na–K–Cl co-transporter 2.

Rats with induced cirrhosis in the current investigation showed significant increase in the renal gene expression of sodium–hydrogen antiporter 1 (NHE-1) and aquaporin-1 compared to normal rats (*p* < 0.001), Fig. 6A. All tested doses of At-rt failed to significantly decrease renal expression of either NHE-1 or aquaporin-1 compared to untreated positive control rats (*p* > 0.05), indicating that this is not the mechanism that accounts for its diuretic effect. On the other hand, all tested doses of As-rt, At-ar and the highest tested dose of As-ar significantly decrease the renal expression of both NHE-1 and aquaporin-1. The extract that typically affected both transporters was As-rt and this effect operated at all dose levels, Fig. 6A.

According to the data obtained from the OPLS model, most biologically active compounds towards NHE-1 were astragaloside VIII, 16-octadecenoic acid methyl ester and orientin and towards aquaporin-1 were hyperoside, 16-octadecenoic acid methyl ester and astragaloside VIII in descending order, Figs. 6B1,2 and S1C. Astragaloside VIII and orientin was detected in all extracts except for At-rt whereas 16-octadecenoic acid methyl ester existed only in As-rt and At-ar. Hyperoside was present only in As-rt*,* supplementary Tables S1 and S3.

#### Extracts acting at the thick ascending limb of the loop of Henle and the distal convoluted tubule:

**Figure 7:**
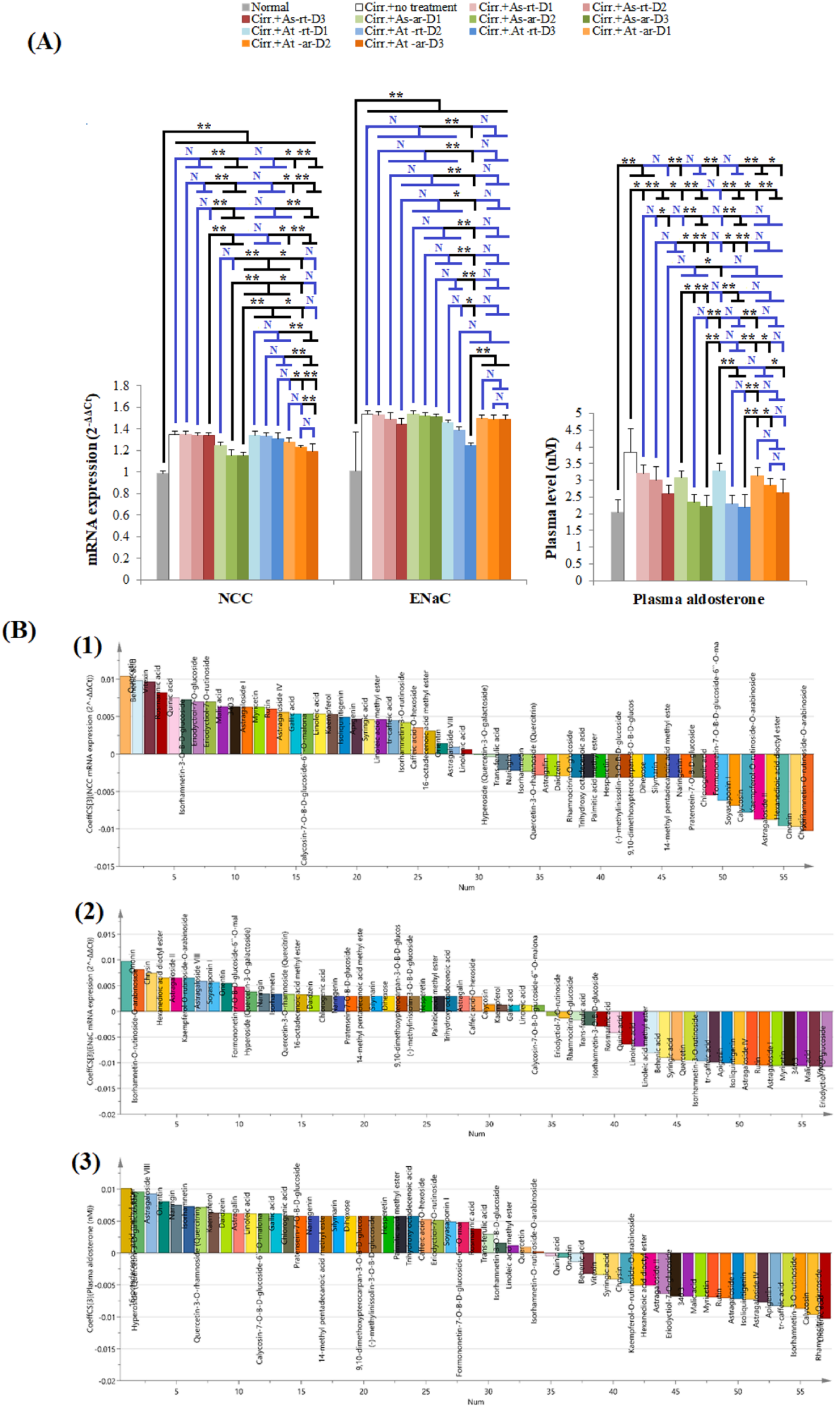
The effect of different *Astragalus* extracts on mRNA expression of the distal convoluted tubule transporters and collecting ducts mechanisms and their coefficient plot of OPLS model. (**A**): Effect of different *Astragalus* extracts on the expression of different renal transporters of the distal convoluted tubule and collecting ducts mechanisms, (**B**): Coefficient plot of OPLS model for diuretic mechanisms; (1) NCC (2) ENaC (3) aldosterone. ANOVA test was used to compare between the different groups with Post Hoc Test (Tukey) to compare different groups. *: Statistically significant at *p* ≤ 0.05, **: Statistically significant at *p* ≤ 0.001, N: Statistically non-significant (*p* > 0.05), n = 8; all results are presented as mean ± SD. (Cirr.: induced cirrhosis by i.p. administration of CCl_4_ (diluted 1:6 with mineral oil) as follows: The first 10 doses were received every 5 days, the subsequent 10 doses were administered every 4 days, and the last 7 doses were given every 3 days, As: *Astragalus spinosus*, At: *Astragalus trigonus*, ar: aerial organs, rt: root organs, D1: low dose, D2: medium dose, D3: high dose, NCC: sodium-chloride symporter and ENaC: epithelial sodium channel.

Rats with CCl_4_-induced cirrhosis showed significantly higher renal expression of both Na–K-Cl co-transporter 2 (NKCC2) and sodium-chloride symporter (NCC) transporters compared to normal negative control rats (*p* < 0.001), Figs. 6A, 7A. Regarding the effect of different extracts on NKCC2, all extracts, except for At-rt, significantly decreased renal expression of NKCC2 with the most prominent effect observed with the highest tested dose of At-ar. Then again, two extracts (As-ar and At-ar) significantly decreased gene expression of NCC at all tested doses, while the two other extracts (As-rt and At-rt) failed to show any significant difference in NCC expression in any tested dose compared to positive control rats. The least decrease in NCC renal expression was observed in As-ar-D3 treated rats.

The top active compounds in reducing the expression of NKCC2 in reference to the OPLS model results were astragaloside VIII, 16-octadecenoic acid methyl ester and hyperoside, Figs. 6B3 and S1C. For NCC, the compounds that decreased its expression to the greatest extent were isorhamnetin-O-rutinoside-O-arabinoside, chrysin and ononin, Figs. 7b1 and S1C.

#### Extracts acting at the collecting ducts:

Our results that revealed significant hyperaldosteronism in cirrhotic rats with plasma aldosterone levels 1.9 folds higher than those observed with normal rats (p < 0.001). Epithelial sodium channel (ENaC) renal expression was significantly elevated in untreated cirrhotic rats compared to normal rats (*p* < 0.001), Fig. 7A. The only extract that significantly reduces ENaC renal expression compared to the positive control rats was the highest tested dose of At-rt.

OPLS model analysis revealed that ENaC expression was mostly decreased by the influence of eriodyctiol-7-O-glucoside (detected in As-rt and At-rt), vitexin (detected in all extracts except for As-ar) and malic acid, Figs. 7B2 and S1C. All of the three top scoring compounds are present in At-rt*,* supplementary Tables S1 and S3. Plasma aldosterone reduction was influenced by the action of linolenic acid (present in As-ar and At-rt), rhamnocitrin-O-glucoside (present only in At-ar) and calycosin (present in all extracts except for As-rt). As-ar and At-rt contain two of the top active compounds against aldosterone and caused lower levels of plasma aldosterone, while As-rt extract lack any of these top active compounds and showed little influence on plasma aldosterone*,* Figs. 7B3, S1C and supplementary Tables S1 and S3.

#### The overall effect of different extracts on diuresis and serum electrolytes

The overall diuretic effect was evaluated by comparing the volume of 24 h collected urine after placing rats of each group in metabolic cages. The rats with untreated cirrhosis showed significantly less collected urine volume compared to normal rats and all treatments succeeded to significantly increase urinary output compared to positive control rats (*p* < 0.001) (data not shown). Upon comparing the mean urinary output of the groups receiving the highest tested dose of each extract, the order was as follows: As-rt D3 ≥ At-ar D3 > As-ar D3 > At-rt D3 with a statistically significant difference between each group and the preceding group except for As-rt and At-ar that showed no statistically significant difference between them (data not shown). This differential effect can be clarified by identifying the dominant diuretic mechanisms for each extract (explained in the next section).

Induction of cirrhosis in the current work caused significant hyponatremia (*p* < 0.001), supplementary Tables S4 As-rt-D3 and At-ar-D3, showed significant reduction in serum sodium level compared to untreated rats (*p* < 0.001), supplementary Tables S4. As-rt-D3 was the only extract that was capable to return serum sodium to its normal levels, as it shows no significant difference compared to normal rats (*p* = 1.00).

The present model of cirrhosis induction was associated with significant decrease in serum potassium level compared to normal rats (*p* < 0.001), supplementary Table S5. At-rt and As-ar showed the greatest potassium levels. The higher doses At-rt showed serum potassium level exceeding the average values in normal rats (*p* < 0.001 for At-rt-D3). On the other hand, rats treated with the higher doses of At-ar, As-ar and all tested doses of As-rt showed no significant difference in serum potassium levels compared to normal rats.

#### Prominent diuretic mechanism of each extract

In order to identify the most prominent mechanism affected by each extract to exert its diuretic effect, the % decrease in mRNA expression of renal transporters and plasma aldosterone upon treatment with the highest tested dose of each extract were compared to the positive control group, Table 1. As-rt-D3 showed the greatest % reduction in proximal tubule transporters; NHE1 and aquaporin-1 compared to other transporters and compared to other extracts. It also showed high percentage reduction in NKCC2 of the thick ascending limb of the loop of Henle. Though As-rt D3 showed significant reduction in plasma aldosterone, this reduction was not reflected on NCC and it slightly affected ENaC. As-ar-D3 caused reduction in aldosterone level by 42.3% that more prominently affected NCC rather than ENaC. It also significantly affected NHE1, aquaporin-1 and NKCC2 but to a much lesser extent than As-rt-D3 or At-ar-D3. Compared to As-rt-D3, At-ar-D3 showed slightly less effect on NHE-1, aquaporin-1 and slightly more effect on NKCC2 renal expression with no statistically significant difference between them (*p* = 0.961, 0.139 and 0.524 respectively). Contrariwise, At-ar-D3 showed much greater effect on the aldosterone sensitive NCC compared to As-rt-D3 (*p* < 0.001). Here the reduction of plasma aldosterone more significantly affected NCC rather than ENaC.

**Table 1:** Metabolites identified in *Astragalus* samples extracts using UPLC-MS in negative ionization mode.

No	Rt (min.)	[M-H]^−^	Element composition	MS^n^ ions m/z (-)	Identified compounds	Chemical class
1	1.10	341.29	C15H18O9	179.15, 135.14	Caffeic acid-O-hexoside	Phenolic acid
2	1.21	191.16	C7H12O6	127.12	Quinic acid	Phenolic acid
3	1.26	133.08	C4H6O5	115, 71	Malic acid	Dicarboxylic acid
4	1.35	169.11	C7H6O5	125.1	Gallic acid	Phenolic acid
5	1.50	353.3	C16H18O9	191.16, 179.15	Chlorogenic acid	Phenolic acid
6	1.60	179.15	C9H8O4	135.14	tr-caffeic acid	Phenolic acid
7	1.69	197.17	C9H10O5	153.16, 182.14	Syringic acid	Phenolic acid
8	1.83	359.31	C18H16O8	197.1, 179.1, 161.13	Rosmarinic acid	Phenolic acid
9	1.88	725.63	C32H38O19	593.52, 447.38, 285.23, 133.12, 151.1	Kaempferol-O-rutinoside-O-arabinoside	Flavonoidal glycoside
10	2.32	755.65	C33H40O20	623.54, 477.4, 315.26, 271.2, 151.1	Isorhamnetin-O-rutinoside-O-arabinoside	Flavonoidal glycoside
11	2.67	341.29	C12H22O11	113.09, 119.1, 161.13, 179.15	Dihexose	Disaccharide
12	2.87	609.51	C27H30O16	301.23, 272.21, 255.2, 151.1	Rutin (quercetin-3-O-rutinoside)	Flavonoidal glycoside
13	10.71	595.53	C27H32O15	449.39, 287.24, 151.1	Eriodyctiol-7-O-rutinoside	Flavonoidal glycoside
14	10.79	623.54	C28H32O16	315.26, 300.22, 271.2, 151.1	Isorhamnetin-3-O-rutinoside	Flavonoidal glycoside
15	11.31	193.18	C10H10O4	178.15,149.17	ferulic acid	Phenolic acid
16	11.93	531.44	C25H24O13	283, 136.11, 211.19, 239.2, 268.22	Calycosin-7-O-B-D-glucoside-6``-O-malonate	Flavonoidal glycoside
17	12.39	515.44	C25H24O12	429.4, 267.26	Formononetin-7-O-glucoside-6``-O-malonate	Flavonoidal glycoside
18	13.33	579.53	C27H32O14	271.25, 151.1, 119.14, 107.09	Naringin	Flavonoidal glycoside
19	13.81	463.37	C21H20O12	301.23, 272.21, 255.2, 151.1	Hyperoside (Quercetin-3-O-galactoside)	Flavonoidal glycoside
20	13.98	447.37	C21H20O11	357.29, 327.27, 285.23	Orientin (Luteolin-8-C-glucoside)	Flavonoidal glycoside
21	14.04	447.37	C21H20O11	285.23, 133.12, 151.1	Astragalin (Kaempferol-3-O-glucoside)	Flavonoidal glycoside
22	14.15	449.39	C21H22O11	287.24, 151.1	Eriodyctiol-7-O-glucoside	Flavonoidal glycoside
23	14.20	477.4	C22H22O12	315.26, 300.22, 271.2, 151.1	Isorhamnetin-3-O-B-D-glucoside	Flavonoidal glycoside
24	14.33	447.37	C21H20O11	301.23, 272.21, 255.2, 151.1	Quercetin-3-O-rhamnoside (Quercitrin)	Flavonoidal glycoside
25	15.91	431.37	C21H20O10	341.29, 311.27, 269.23	Vitexin (Apigenin-8-C-glucoside)	Flavonoidal glycoside
26	18.44	461.4	C22H22O11	299.26	Rhamnocitrin-O-glucoside	Flavonoidal glycoside
27	20.16	461.4	C22H22O11	299.26	Pratensein-7-O-B-D-glucoside	Flavonoidal glycoside
28	21.21	429.4	C22H22O9	267.26	Ononin (Formononetin-7-O-glucoside)	Flavonoidal glycoside
29	21.36	461.44	C23H26O10	299.3	9,10-dimethoxypterocarpan-3-O-B-D-glucoside	Flavonoidal glycoside
30	21.53	461		299.1, 268.8	(-)-methylinissolin-3-O-B-D-glucoside	Flavonoidal glycoside
31	21.89	317.23	C15H10O8	179.11, 151.1, 137.11	Myricetin	Flavonoidal aglycone
32	22.01	301.23	C15H10O7	272.21, 255.2, 151.1	Quercetin	Flavonoidal aglycone
33	22.11	481.43	C25H22O10	301.2, 453.42	Silymarin	Flavonolignans
34	22.30	271.25	C15H12O5	151.1, 119.14, 107.09	Naringenin	Flavonoidal aglycone
35	22.69	301.27	C16H14O6	286.24, 164.12, 136.15	Hesperetin	Flavonoidal aglycone
36	22.91	253.23	C15H10O4	209.22, 135.1	Daidzein	Flavonoidal aglycone
37	23.19	255.25	C15H12O4	135.1, 119.14	Isoliquiritigenin	Chalcone derivative
38	23.45	285.23	C15H10O6	133.12, 151.1	Kaempferol	Flavonoidal aglycone
39	23.69	269.23	C15H10O5	225.22, 151.1, 117.13	Apigenin	Flavonoidal aglycone
40	24.10	315.26	C16H12O7	300.22, 271.2, 151.1	Isorhamnetin	Flavonoidal aglycone
41	24.56	253.23	C15H10O4	209.22, 151.1	Chrysin	Flavonoidal aglycone
42	28.25	283.26	C16H12O5	136.11, 211.19, 239.2, 268.22	Calycosin	Flavonoidal aglycone
43	28.39	783.96	C41H68O14	651.85, 621.82, 489.71, 179.15, 161.13, 149.12, 131.11	Astragaloside IV	Cycloartane saponin
44	29.05	826	C43H70O15	764.94, 632.83, 88.7	Astragaloside II	Cycloartane saponin
45	29.43	942.12	C48H78O18	795.98, 633.84, 439.7, 421.68	Soyasaponin I	Oleanane saponin
46	30.04	868.04	C45H72O16	824.99, 781.95, 692.88, 662.85, 488.7	Astragaloside I	Cycloartane saponin
47	32.43	912.09	C47H76O17	868.08, 647.87	Astragaloside VIII	Cycloartane saponin
48	33.88	277.42	C18H30O2	259.4	Linolenic acid	polyunsaturated omega-3 fatty acid
49	34.03	329.45	C18H34O5	171.21	9,10,13-trihydroxy-11-octadecenoic acid	monounsaturated fatty acid
50	34.18	269.44	C17H34O2	238.41, 74.08	Palmitic acid methyl ester	saturated fatty acid ester
51	34.53	269.44	C17H34O2	238.41, 74.08, 43.09	14-methyl pentadecanoic acid methyl ester	saturated fatty acid ester
52	34.75	279.44	C18H32O2	261.42	Linoleic acid	polyunsaturated omega-6 fatty acid
53	34.93	293.47	C19H34O2	279.44, 261.42	Linoleic acid methyl ester	polyunsaturated omega-6 fatty acid ester
54	35.00	295.48	C19H36O2	264.45, 221.4, 74.08, 55.1	16-octadecenoic acid methyl ester	monounsaturated fatty acid ester
55	35.79	369.56	C22H42O4	55.1	Hexanedioic acid dioctyl ester	saturated fatty acid diester
56	36.11	339.58	C22H44O2	321.56, 295.57	Behenic acid	saturated fatty acid

At-rt-D3 failed to significantly decrease the expression of most renal transporters, namely NHE-1, aquaporin-1, NKCC2 or NCC (*p* = 0.443, 0.946, 1.000 and 0.846, respectively) compared to untreated rats with induced cirrhosis. This extract also showed the greatest reduction of plasma aldosterone that more significantly affected ENaC rather than NCC and was accompanied with hyperkalemia, supplementary Table S5.

### Effect of Different *Astragalus* Extracts on Portal Hypertension Markers

**Figure 8:**
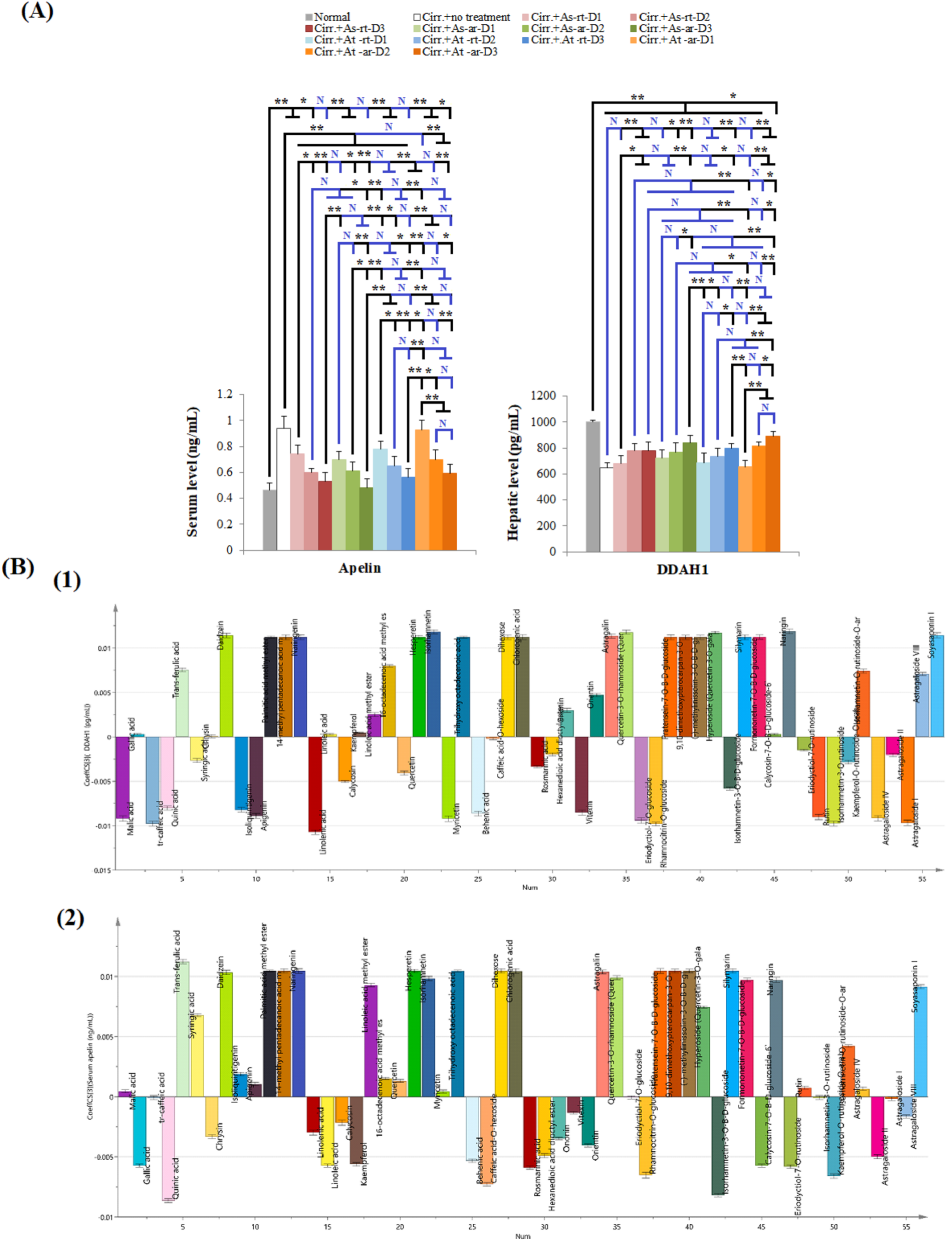
The effect of different *Astragalus* extracts on portal hypertension markers and their coefficient plot of OPLS model. (**A**): Effect of different *Astragalus* extracts on PH markers, (**B**): Coefficient plot of OPLS model for PH markers; (1) apelin (2) DDAH-1. ANOVA test was used to compare between the different groups with Post Hoc Test (Tukey) to compare different groups. *: Statistically significant at *p* ≤ 0.05, **: Statistically significant at *p* ≤ 0.001, N: Statistically non-significant (*p* > 0.05), n = 8; all results are presented as mean ± SD. (Cirr.: induced cirrhosis by i.p. administration of CCl_4_ (diluted 1:6 with mineral oil) as follows: The first 10 doses were received every 5 days, the subsequent 10 doses were administered every 4 days, and the last 7 doses were given every 3 days, As: *Astragalus spinosus*, At: *Astragalus trigonus*, ar: aerial organs, rt: root organs, D1: low dose, D2: medium dose, D3: high dose, DDAH1: dimethylarginine dimethylaminohydrolase 1.

In the present study, the circulating level of apelin was significantly increased in rats with induced cirrhosis compared with healthy rats (*p* < 0.001). With exception for At-ar-D1, all tested doses of all extracts significantly decreased serum apelin compared to rats with untreated induced cirrhosis. The least mean apelin value was observed in sera of rats treated with As-ar-D3, however, this mean value was not statistically different from those of the highest tested dose of other extracts. Hepatic DDAH-1 was significantly decreased in rats with cirrhosis compared to normal rats (*p* < 0.001). High doses and some medium doses of different extracts were able to significantly increase hepatic DDAH-1, Fig. 8A. The highest mean hepatic DDAH-1 was observed in At-ar-D3 treated rats which was significantly higher than As-rt-D3 and At-rt-D3 (*p* = 0.007 and 0.042, respectively), but showed no statistically significant difference with As-ar-D3 (*p* = 0.897).

According to the OPLS model, apelin is more significantly reduced in response to quinic acid (detected in all extracts), isorhamnetin-3-O-B-D-glucoside (present in all extracts except for As-ar) and caffeic acid-O-hexoside (present only in At-ar)*,* Figs. 8B1, S1D and supplementary Tables S1 and S3. The compounds that revealed the greatest ability to increase the level of hepatic DDAH-1 were naringenin, isorhamnetin and quercetin-3-O-rhamnoside (quercitrin)*,* Figs. 8B2, S1D, that were all detected in At-ar (supplementary Tables S1 and S3).

## Discussion

Though widespread study on the chemical content of *Astragalus* extracts and their therapeutic effectiveness, no systematic chemical profiling of *A. spinosus* and *A. trigonus* roots and aerial parts bioactive phytoconstituents has been published to the authors’ knowledge. The roots and aerial extracts of the two species were first chemically profiled using the ultra-high performance liquid chromatography/triple quadrupole mass spectrometry method (UHPLC-QqQ-MS), with the aim of establishing a correlation between the bioactive elements of the extracts and their in vivo alleviation of liver cirrhosis and associated PH.

Metabolites annotation was achieved via comparison of retention times to references and examination of MS data (quasi-molecular ions and fragmentation patterns) of the detected peaks.

The MS spectrum of compound **1** showed a molecular ion peak at m/z 341.29 which was tentatively characterized as caffeic acid-O-hexoside, based on the loss of hexoside (−162.14 Da) and CO_2_ (−44.01 Da) moieties and the presence of characteristic fragments at m/z 179.15 [M–H-hexoside]^−^ and m/z 135.14 [M-H-hexoside-CO_2_]^−^^34^. Furthermore, the MS spectra of compounds **4**, **6**, **7** and **15** displayed base peak ions [M-H]^−^ at m/z 169.11, 179.15, 197.17, and 193.18, respectively in addition to their characteristic decarboxylated fragments. For compounds **7** and **15** another fragment arising from elimination of methyl group (15.03 Da) was readily observed in their MS^2^ spectra at m/z 182.14 and 178.15, respectively. The compounds were accordingly identified as gallic acid (compound **4**), caffeic acid (compound **6**), syringic acid (compound **7**), and ferulic acid (compound **15**), respectively^34,35^. Meanwhile, compound **5** exhibited an intense base peak [M–H]^−^ at m/z 353.3 with diagnostic fragments at m/z 179.15 and m/z 191.16 due to caffeic acid and quinic acid, respectively and thus compound **5** was assigned as monocaffeoylquinic acid (chlorogenic acid) in accordance with the literature^35^, which indicates that caffeic acid is frequently associated with quinic acid. The MS/MS spectrum and fragmentation pattern of chlorogenic acid is depicted in supplementary Fig. S2. Next to the bound ester form of quinic acid (chlorogenic acid), quinic acid was detected in the free acid form as represented by compound **2** which showed an intense base peak [M-H]^−^ ion at m/z 191.16.

Compound **8** generating a molecular ion peak [M–H]^−^ at m/z = 359.31 and a base peak at m/z = 161.13 was identified as rosmarinic acid. The identification was based on the diagnostic product ions observed at m/z 197.17 and 179.15 of the two main constituents of rosmarinic acid: 2-hydroxyl derivative of hydro caffeic acid and caffeic acid, respectively. This pattern of fragmentation was compared with those data reported previously^35,36^.

Malic acid (compound **3**) was suggested for the precursor ion at m/z 133.08 which in turn gave fragments at m/z 115.06 and 71.05 due to successive loss of H_2_O and CO_2_^35^.

Compound **11** was assigned as dihexose based on its parent ion at m/z 341.29, accompanied by daughter peaks at m/z 179.15, corresponding to deprotonated monosaccharide, and at m/z 161.13, due to neutral loss of a monosaccharide molecule. Whereas ions at m/z 119.1 and 113.09, were derived respectively from m/z 179.15 and 161.13 by neutral loss of 2-hydroxyacetaldehyde (60.05 Da) and formaldehyde + water (48.04 Da)^37,38^.

Kaempferol-O-rutinoside-O-arabinoside (compound **9**) and astragalin (compound **21**) produced a characteristic fragment ion at m/z 285.23 due to loss of C_17_H_28_O_13_ and C_6_H_10_O_5_, respectively. These compounds together with kaempferol (compound **38**) showed fragment ions at m/z 151.1 and 133.12 due to Retro Diels–Alder (RDA) reaction^34,39^. Similarly, isorhamnetin-O-rutinoside-O-arabinoside (compound **10**), isorhamnetin-3-O-rutinoside (compound **14**) and isorhamnetin-3-O-B-D-glucoside (compound **23**) showed a distinct fragment ion at m/z 315.26 due to elimination of C_17_H_28_O_13_, C_12_H_20_O_9_ and C_6_H_10_O_5_, respectively. All these compounds and isorhamnetin (compound **40**) showed fragment ions at 300.22 and 271.2 due to loss of [CH_3_]^−^ and [CH_3_ + CHO]^−^, respectively. They also showed the diagnostic RDA fragment at m/z 151.1^34,40^. The MS/MS spectrum and fragmentation pattern of isorhamnetin-3-O-rutinoside is depicted in supplementary Fig. S3. Furthermore, Rutin (compound **12**), hyperoside (compound **19**) and quercitrin (compound **24**) generated a characteristic fragment ion at m/z 301.23 due to removal of C_12_H_20_O_9_, C_6_H_10_O_5_, C_6_H_10_O_4_, respectively. These compounds together with quercetin (compound **32**) produced fragment ions at m/z 272.21 and 255.2 due to loss of [CHO]^−^ and [CO + H_2_O]^−^, respectively. All compounds also produced Retro Diels Alder (RDA) fragment ion at m/z 151.1^35,41^.

Peak **22** at m/z 449.39 [M-H]^−^ was characterized as eriodyctiol-7-O-glucoside. It displayed daughter ion peaks in the MS/MS spectrum at m/z 287.24, due to loss of glucosyl moiety, and at m/z 151.1 arising from a distinct RDA ion. Whereas, Peak **13** with [M-H]^−^ ion at m/z 595.53 showed the same fragmentation pattern of peak **22** (Eriodyctiol-7-O-glucoside) in addition to 146.14 Da (rhamnosyl moiety). This indicated that compound **13** is Eriodyctiol-7-O-rutinoside^40,42^.

Peak **28** was assigned as formononetin-7-O-glucoside (ononin). This identification was based on the daughter ion peak at m/z 267.26 which corresponds to [formononetin-H]^−^^43^. The [M-H]^−^ ion at m/z 429.4 with additional 162.14 Da indicated that formononetin was attached to a glucosyl moiety. Compound **17** was fragmented in the same way as compound **28** (formononetin-7-O-glucoside) with an extra 86.05 Da corresponding to a malonyl moiety. This assured that compound **17** is formononetin-7-O-glucoside-6``-O-malonate^43^.

Naringin (compounds **18**) and naringenin (compounds **34**) were fragmented similarly except that naringin had an extra 308.28 Da corresponding to a disaccharide moiety. Both compounds showed RDA fragments at m/z 151.1 and 119.14, and a fragment at 107.09 corresponding to [151.1-CO_2_]^−^^35,43^.

Compound **42** with [M-H]^−^ ion at m/z 283.26 was identified as calycosin. It showed fragment ions at m/z 268.22, 239.2 and 211.19 due to [M-H-CH_3_]^−^, [M-H-CH_3_-CHO]- and [M-H-CH_3_-CHO-CO]^−^, respectively. It also showed a fragment ion at m/z 136.11 due to RDA reaction. Compound **16** was fragmented in the same way as calycosin with the addition of 248.19 Da due to glucoside-malonate moiety. Therefore, compound **16** was identified as calycosin-7-O-glucoside-6``-O-malonate^43^.

Different fragmentation patterns were observed in MS/MS experiments of flavone-C glycosides. Losses of 120.1 and 90.08 Da were observed, corresponding to cross-ring cleavages in the sugar unit^40^. This was observed in the mass spectrum of orientin (compound **20**) and vitexin (compound **25**) which showed parent ions [M−H]^−^ at m/z 447.37 and 431.37, respectively. Upon fragmentation, they produced fragments ions at m/z 357.29 and 341.29 [M-H-90.08]^−^ and 327.27 and 311.27 [M-H-120.1]^−^, respectively. They also produced fragment ions due to flavonoidal aglycones at 285.23 [luteolin-H]^−^ and 269.23 [apigenin-H]^−^, respectively.

Compounds **26**, **27**, **29** and **30** giving rise to deprotonated ions at m/z 461.4 and fragment ions in their MS^2^ spectra at m/z 299.26 [M-162.14]^−^, due to loss of a glycosyl moiety, were identified as rhamnocitrin-O-glucoside, pratensein-7-O-B-D-glucoside, 9,10-dimethoxypterocarpan-3-O-B-D-glucoside and (-)-methylinissolin-3-O-B-D-glucoside, respectively^43,44^.

In accordance with literature^35^, compound **31** was identified as myricetin. This was confirmed by [M-H]^−^ ion at m/z 317.23 and daughter ion peaks in the MS/MS spectrum at m/z 179.11, 151.1 and 137.11. These daughter ions are typical of retro Dies-Alder (RDA) reactions of flavon-3-ols having a dihydroxylated A ring, and m/z 137.11 is a typical fragment of the trihydroxylated B ring.

Within the same context, silymarin isomers (compound **33**) were identified by the precursor ion at m/z 481 with distinct fragment at (m/z 301.23) formed by the loss of a coniferyl alcohol unit as illustrated in Table 2^45,46^.

**Table 2:** % decrease in mRNA expression of renal transporters and plasma aldosterone upon treatment with the highest tested dose of each extract compared to positive control group.

	NHE1 (%)	Aquaporin-1 (%)	NKCC2 (%)	NCC (%)	ENaC (%)	Aldosterone (%)
As-rt D3	21.62	29.44	13.57	0.74	6.49	32.38
As-ar D3	10.27	7.22	9.29	14.81	1.95	42.30
At-ar D3	17.30	26.11	16.43	11.85	3.25	31.33
At-rt D3	1.08	2.22	1.43	2.96	18.83	43.08

As: *Astragalus spinosus*, At: *Astragalus trigonus*, ar: aerial organs, rt: root organs, D3: high dose, NKCC 2: Na–K-Cl co-transporter 2, NCC: sodium-chloride symporter, NHE1: sodium–hydrogen antiporter 1 and ENaC: epithelial sodium channel.

Compounds **36**, **39** and **41** displaying intense [M−H]^−^ ions at m/z 253.23, 269.23 and 253.23 were identified as daidzein, apigenin and chrysin, respectively, where they all underwent fragmentations by loss of CO_2_ (− 44.01 Da) confirmed by previous literature data^35,43^. Chrysin and daidzein were differentiated by the diagnostic RDA fragment which was at m/z 151.1 in case of chrysin, indicating a dihydroxylated A ring, and at m/z 135.1 in case of daidzein, indicating a monohydroxylated A ring.

Hesperetin was proposed for compound **35** which exhibited a molecular ion [M−H]^−^ at m/z 301.27 and fragmented to m/z 286.24 [M-H-CH_3_]^−^, and m/z 164.12 and 136.15, which were arised from RDA reaction related the cleavage of bonds 1 and 2 of C ring^35,47^.

Compound **37** gave rise to a base peak [M-H]^−^ ion at m/z 255.25 and structurally informative RDA product ions at m/z 135.1 and 119.14. That was consistent with the structure of isoliquiritigenin^48,49^.

Compounds **43–47** were identified comparing their high-performance liquid chromatography (HPLC) elution order, MS, and MS/MS data with those previously reported^43,50,51^.

Full MS spectrum of compound **43** displayed a deprotonated molecule [M−H]^−^ at m/z 783.96; MS/MS spectrum showed ions at m/z 651.85 [M-H-132.11]^−^, 621.82 [M-H-162.14]^−^, and 489.71 [M-H-162.14-132.11]^−^ due to the loss of a glucopyranosyl unit followed by one xylopyranosyl moiety. The fragmentation also produced a number of specific ions, such as the sugar ions at m/z 179.15 [Glu − H]^−^ , 161.13 [Glu − H − H_2_O]^−^ , 149.12 [Xyl − H]^−^ and 131.11 [Xyl − H − H_2_O]^−^ , which confirmed the presence of glucose and xylose. Therefore, compound **43** was assigned as astragaloside IV.

Meanwhile, MS/MS analysis of an ion peak at m/z 826 [M−H]^−^ proved the loss of one acetyl group, 764.94 [M-H-(Ac-H_2_O)]^−^, followed by one xylopyranosyl moiety, 632.83 [M-H-(Ac-H_2_O)-(Xyl-H_2_O)]^−^, and one glucopyranosyl moiety, 488.7 [M-H-(Ac-(Xyl-H_2_O)-(Glu-H_2_O)]^−^. The peak **44** was identified as astragaloside II.

Furthermore, The MS of peak **46** showed an [M−H]^−^ ion at m/z 868.04 and MS/MS ion spectrum indicated the presence of an astragaloside aglycone (m/z 488.7) together with two acetyl moieties, 824.99 [M-H-Ac]^−^, 781.95 [M-H-2Ac]^−^, xylose, 692.88 [M-H-Ac-(Xyl-H2O)]^−^ and glucose moieties, 662.85 [M-H-Ac-(Glu-H_2_O)]^−^. Therefore, peak **46** was assigned as astragaloside I.

Within the same context, the deprotonated ion [M-H]^−^ at m/z 912.09 was ascribed to astragaloside VIII. MS/MS spectra of this compound indicated that it had a carboxyl group, 868.08 [M-H-CO_2_]^−^, and at least two xylose moieties, 647.87 [M-H-2(Xyl-H_2_O)]^−^.

Compounds **43, 44, 46 and 47** are cycloartane saponins. The example of oleanane saponins identified in this study is soyasaponin I represented by compound **45**. The identification of soyasaponin I was based on its deprotonated ion at m/z 942.12[M-H]^−^, fragment ions at m/z 795.98 [M-H-146.14]^−^, 633.84 [M-H-146.14-162.14]^−^, 439.7 [M-H-146.14-162.14-194.14]^−^, 421.68 [M-H-146.14-162.14-194.14-18.02]^−^ and literature data^43^. The neutral loss of 146,0.14 162.14 and 194.14 Da allowed the identification of rhamnopyranosyl, galactopyranosyl and glucouronic acid moieties, respectively.

Compound **52** generating a molecular ion [M–H]^−^ at m/z 279.44 and a fragment ion in MS/MS spectrum at m/z 261.42 due to loss of H_2_O, was identified as linoleic acid, while compound **53** with an extra 15.03 Da was characterized as linoleic acid methyl ester. Meanwhile, compound **48** producing molecular ion at m/z 277.42 and a fragment ion at m/z 259.4, which are 2 Da lower than those of linoleic acid, was proposed to be linolenic acid^34,52^.

Furthermore, compound **49** produced a deprotonated ion at m/z 329.45 was identified as 9,10,13-trihydroxy-11-octadecenoic acid. In MS^2^ spectrum, a fragment ion at m/z 171.21 was observed due to cleavage of the bond between C9 and C10^37^.

Compounds **50** and **51** having their molecular ion peaks at m/z 269.44 were identified as palmitic acid methyl ester and 14-methyl pentadecanoic acid methyl ester. Both compounds exhibited similar fragment ions in their MS^2^ spectra, where fragment ion at m/z 238.41 [M-31.03]^−^ represented loss of a methoxy group confirming a methyl ester compounds. Ion at m/z 74.08 is the McLafferty rearrangement ion, also a specific ion confirming that the spectra are that of methyl esters^34^. 14-methyl pentadecanoic acid methyl ester had an additional ion at m/z 43.09 due to loss of isopropyl group^34^.

Within the same context, compounds **54** and **55** giving rise to deprotonated ions at m/z 295.48 and 369.56 were proposed to be 16-octadecenoic acid methyl ester and hexanedioic acid dioctyl ester, respectively. Upon fragmentation, both compounds generated a base peak at m/z 55.1 due to loss of the hydrocarbon ion C_4_H_7_^−^ . Compound **54** showed fragment ions at 264.45 [M- 31.03]^−^ due to loss of methoxy group, and at m/z 221.4 corresponding to loss of McLafferty ion which is characteristic to methyl esters^34^. Meanwhile, compound **56**, with a respective mass of m/z 339.58 [M−H]^−^ , was annotated as behenic acid with MS^2^ fragment ions appearing at m/z 321.56 and 295.57 suggestive for the loss of H_2_O and CO_2_, respectively^52^.

Very few studies explored the hepatoprotective potential or antifibrotic activities of *A. spinosus* and *A. trigonus.* Two studies supported *A. spinosus* hepatoprotective potential; the first revealed that saponin glycosides in *A. spinosus* roots could modulate CCl_4_-induced toxicity and lethality in liver^53^. The other was conducted on rabbits where the daily administration of *A. spinosus* root extract to rabbits with hydrogen peroxide-induced oxidative stress showed significant improvement in liver sections upon histopathological examination^54^. To our knowledge, this is the first study to examine the effect of *A. spinosus* aerial parts and *A. trigonus* root and aerial parts extracts on cirrhosis and its complications with mechanistic insight of the role of identified phytoconstituents.

Liver cirrhosis can be sensitively assessed using a combination of multiple direct and indirect biomarkers. Usually, direct markers reflect fibrogenesis process and ECM turnover whereas indirect biomarkers indicate liver functionality alterations of hepatic function^55^. Herein, hepatic levels of TGF-β and RXR-α were determined as direct biomarkers reflecting HSCs activation and proliferation, while ECM turnover was evaluated by assessing hepatic MMP-2. Hepatocellular damage and alterations in liver functions were estimated by determining serum level of ALT, AST and albumin.

TGF-β is a key profibrogenic mediator involved in all fibrogenesis processes starting from initiation of inflammation-induced liver injury to development of fibrosis and cirrhosis ending to hepatocellular carcinoma. Elevated levels of TGF-β augment hepatocyte destruction and facilitate HSCs activation and ECM deposition^4^. Though effect of other *Astragalus* species, such as *A. membranaceus, A. trojanus and A. oleifolius,* on TGF-β in different conditions was formerly examined^56–58^ , influence of *A. spinosus* and *A. trigonus* on TGF-β was not studied before. OPLS model revealed that the top efficient compounds modulating hepatic TGF-β and reducing its level were linoleic acid methyl ester, ferulic acid and naringin. It was proved earlier that circulating plasma linoleic acid in patients with simple hepatic steatosis, the progressive nonalcoholic steatohepatitis or any end-stage liver diseases were significantly lower than in healthy subjects, hence, plasma fatty acids level may serve as a prognostic marker in cirrhosis^59,60^. Thus, administration of extracts containing this essential fatty acid may compensate for cirrhosis-associated linoleic acid and its derivatives deficiency. Furthermore, upon comparing the involvement of different dietary lipids on the modulation of TGF-β in the platelets, it was found that platelets lysates treated with corn oil, composed primarily of linoleic acid, showed the least levels of TGF-β^61^. Ferulic acid potently improved hepatic fibrosis via inhibition of the TGF-β1/Smad pathway in vitro and in-vivo^62^. Yet, naringenin was proved to inhibit TGF-β ligand–receptor interaction which is the initial step of TGF-β signaling^63^. Consequently, naringenin exerted potent antifibrotic effect in ameliorating hepatic, cardiac and lung fibrosis via its action on TGF-β pathways^64–66^. Since, among the top efficient compounds modulating hepatic TGF-β, As-ar contains only linoleic acid methyl ester, this means that this compound accounts for the activity of As-ar towards TGF-β to a great extent and is present in high concentration within this extract.

MMP-2 is a key fibroproliferative marker in chronic liver diseases with high diagnostic accuracy^67^. It degrades the excessively produced ECM during fibrogenesis^55^. Hepatic MMP-2 levels modulation was mostly affected by linolenic acid, tr-caffeic acid and isorhamnetin-3-O-rutinoside according to the OPLS model. Previous reports strongly agree with the correlation between these three compounds and MMP-2. For example, Hubbard et al., proved that linolenic acid administration could decrease MMP-2 levels in isolated mammary tumors from mice, .besides, it could potently inhibit MMP-2 activity^68^. Further, Eleonora et al., revealed that linolenic acid protective role in periodontal diseases could be the result of being an inhibitor to MMP-2 and MMP-9^69^. Caffeic acid was reported to down regulate MMP-2 gene expression and suppress MMP-2 enzyme activity in HepG2 cells^70^. Potent MMP-2 enzyme inhibition was also observed with caffeic acid derivatives such as esters and amides^71,72^. Furthermore, isorhamnetin-3-O-rutinoside is a polyphenolic flavone found in many herbal species that can depress MMP-2 levels in various conditions^73,74^. The combined presence of these top scored compounds affecting MMP-2 in As-ar and At-rt may explain the observed less hepatic MMP-2 levels in rats treated with these extracts than other treated groups.

Vitamin A regulates many physiological processes, including epithelial growth and immunological processes. Vitamin A pleiotropic effects are exerted either by vitamin A itself or by the main active metabolite, all-trans retinoic acid. They regulate the expression of a battery of target genes through several nuclear receptors such as RARs, RXRs, and PPARβ/δ^75^. Vitamin A is primarily stored in HSCs that lose vitamin A storage capacity during active liver injury and convert into activated ECM-producing myofibroblasts, which contributes to hepatic fibrosis then cirrhosis^76^. Consequently, the mRNA expression levels of RARβ and RXR-α are down-regulated in cirrhotic patients, suggesting the major role of these nuclear receptors in HSC activation and liver fibrogenesis^77^. Restoration of RAR/RXR levels suppresses liver fibrosis by blocking HSCs activation and proliferation^5^. As shown by OPLS model, activity on RXR-α is accredited mainly to isorhamnetin-O-rutinoside-O-arabinoside, soyasaponin I and formononetin-7-O-glucoside-6``-O-malonate. It was observed that extracts especially containing isorhamnetin-O-rutinoside-O-arabinoside caused significant elevation in RXR-α level which points to the key role of in restoring vitamin A storage capacity within the liver. In a number of studies, isorhamnetin suppressed HSCs activation via inhibition of TGF-β/Smad signaling pathway^78,79^. Suppression of HSCs activation is usually accompanied by restoration of their vitamin A storage capacity and subsequent upregulation of RXR^80^. The presence of glycoconjugates to isorhamnetin in the isorhamnetin-O-rutinoside-O-arabinoside compound may result in intensifying its ability to suppress HSCs activation, as in many cases it was observed that glycoconjugated derivatives are more active than the parent compound^81^.

ALT and AST are normally present in the hepatocyte cytosol. They serve as sensitive markers for evaluating the liver functional status in hepatic diseases as increased circulating ALT and AST level reflects cell damage and leakage^82^. ALT is predominantly found in hepatocyte cytosol whereas AST is found within the cytosol and mitochondria of the hepatocytes as well as many other organs^83^. Though ALT is more accurate than AST as hepatocyte injury marker, AST is more indicative for hypoxic and toxic damage, because zone 3 of the acinus with higher AST concentration is more susceptible to toxic damage^84^. On the other hand, albumin is synthesized in the liver and is an indicator of liver function^85^. Induction of cirrhosis was coupled with many folds increase in ALT and AST serum levels which indicates significant loss of hepatocytes integrity as well as liver synthetic function as a consequence of cirrhosis. Effect of different extracts was more pronounced on ALT than AST reflecting the liver-specific injury reversal by *Astragalus* species. Amongst all treated groups, the least ALT, AST and highest albumin levels were observed with the highest tested dose of As-ar. This result matches the observed greatest ability of this extract to regress hepatic cirrhosis, indicated by the highest significant reduction of TGF-ꞵ and MMP-2, and reversal of HSCS activation, marked by restoration of HSCs vitamin A storage capability and RXR expression level. Fibrosis reduction led to hepatocytes repair and increasing hepatic synthetic capabilities. As-ar D3 revealed significantly less AST levels compared to all other treated groups which reflects a potent ability of the arial parts of *Astragalus spinosus* to repair hypoxic damage and may indicate the presence of significant amount of antioxidant compounds in their extracts.

Top detected compounds scoring activities against AST and ALT using OPLS model were identical to each other and to those of RXR. Whilst the top scoring compounds against albumin was ononin, followed by isorhamnetin-O-rutinoside-O-arabinoside and soyasaponin I that were the top scoring compounds against ALT, AST and RXR. The coincidence of the top scoring compounds that were able to restore RXR, hepatocytes integrity and synthetic function implies a strong correlation between hepatic vitamin A content and hepatic regeneration. In support of this conclusion, a study was done to test the effect of vitamin A deficiency on hepatic regeneration in male and female rats after partial hepatectomy. This study proved that vitamin A is essential for the survival of hepatocytes^86^. Therefore, compounds capable of restoring vitamin A content with subsequent elevation of RXR, would restore hepatocytes integrity and synthetic function as indicated by the significant decrease in ALT and AST and increase in albumin synthesis.

Cirrhosis is associated with altered sodium and water metabolism regulation. During the course of cirrhosis, progressive impairment of kidney ability to maintain the volume of extracellular fluid within normal limits occurs. This is a consequence of an abnormal increase in tubular sodium and kidney disability to adjust the amount of sodium excreted. As a result, accumulation of solute-free water and an increase in total body water relative to total sodium content follows^87^. Diuretics are a heterogenous group of medications that function to control sodium and water metabolism in various sites within the renal tubules via various mechanisms of action. The use of diuretics is one of the widely used strategies to control PH and edema in cirrhotic patients^88,13^.

The proximal convoluted tubule is the site where the majority of sodium is reabsorbed. Within the proximal convoluted tubule, NHE-1 is probably the most important isoform due to its multi-functionalities unlike other isoforms such as NHE-3 that carries out the sole function of mediating the renal sodium absorption. For example, NHE-1 regulates intracellular pH, cell volume and intracellular sodium homeostasis^89,90^. NHE-1 may also modulate other transporters, such as apical membrane NHE-3, possibly through actin cytoskeleton remodeling, as well as Na^+^–K^+^ ATPase through functional interaction with these transporters^90,91^. NHE-1 has been also shown to regulate cell cycle, proliferation, migration and adhesion, resistance to apoptosis and to implicate hypertension pathogenesis^91,92^. Thus NHE-1 downregulation can result in diuresis and relief of PH. Aquaporin-1 is the principal water-transporter in cell plasma membranes in both apical and basolateral membranes of kidney proximal tubule, where it facilitates > 70% of transepithelial water transport and causes urine concentration^15^. Aquaporin-1 has been shown to affect cell migration, cell proliferation, and angiogenesis. Aquaporin-1 inhibition or downregulation causes significant urinary concentrating defect with subsequent diuretic effect in many tested diuretics^93^. In the current investigation, induction of cirrhosis was associated with significant increase in both transporters. In accordance, the abundance of aquaporin-1 was formerly detected in the kidneys of rats with CCl_4_-induced cirrhosis, pointing to potentially vital effects of having cirrhosis on the proximal tubules. On the other hand, renal NHE-1 was not previously detected in rats with induced cirrhosis, yet, hepatic NHE-1 up regulation was found to contribute to the pathogenesis of liver fibrotic diseases^94^. These effects may account for the sodium retention and increased fluid absorption in this segment during cirrhosis progression^95^. According to data obtained from OPLS model, both NHE-1 and aquaporin-1 share the two active compounds astragaloside VIII and 16-octadecenoic acid methyl ester indicating that the presence of either of these compounds may be essential for selective activity at the proximal tubules. Since At-rt lack the presence of any of the top scoring compounds for either NHE-1 or aquaporin-1, it does not show any effect on the expression of these transporters. Though no previous study was done to show the diuretic effect of the claimed compounds in their purified form, several studies proved diuretic effect of plant extracts containing these compounds^96,97^.

The thick ascending limb of the loop of Henle accounts for a total of 20–25% of the filtered NaCl reabsorption. The major apical entry pathway for Na^+^ is provided by Na–K-Cl co-transporter (NKCC2), which accounts for ∼80% of the total Na^+^ reabsorption in this region of the nephron^98^. This area of the nephron is the site of action of loop diuretics where they bind to NKCC and inhibit its action. Inhibition of NKCC2 or its diuretic effect results in impairing reabsorption of Na^+^, K^+^, and Cl^−^ and delivering an increase in Na^+^ to the distal tubule. These changes decrease the osmotic driving force with subsequent diuretic effect^14^. At the distal convoluted tubule, reabsorption of 5–10% of glomerular filtrate occurs by the action of Na-Cl co-transporter (NCC)^99^. Blockade of NCC, which is the mechanism of action of thiazide diuretics, or its down regulation result in increased Na^+^ delivery to collecting ducts, enhanced K^+^ wasting and diuresis^14^. Induction of cirrhosis using CCl4 significantly increased renal expression of NKCC2 and NCC. This is in accordance with previous reports that proved significant increased renal abundance of sodium transporter proteins, including NKCC2 and NCC, and sodium retention in cirrhotic rats^95^. Top active compounds in reducing NKCC2 expression were also identical to the top scoring active compounds that decrease aquaporine-1 expression. Thus, similar activities of different extracts towards both transporters were obtained. Also, two compounds (astragaloside VIII and 16-octadecenoic acid methyl ester) were common with the top scoring compounds for reducing NHE-1 expression. For NCC, isorhamnetin-O-rutinoside-O-arabinoside, the compound that reduced the expression for the greatest extent was also the compound that mostly decreased ALT and AST, besides ononin was the top compound responsible for the restoration of albumin synthetic function which indicates a very close association between cirrhosis degree and the NCC renal expression. Thus, the extracts of the arial parts of the tested *Astragalus* species, especially As-ar, that showed greater effect on restoring hepatocytes integrity and synthetic function induced diuresis mainly by affecting NCC expression.

Collecting ducts possess two mechanisms that contribute to sodium reabsorption control; the epithelial Na^+^ channel (ENaC) and aldosterone via binding to aldosterone receptor. Since only 3% of the filtered Na ^+^ is reabsorbed at the collecting duct, drugs inhibiting or down regulating ENaC or aldosterone receptor do not result in appreciable diuresis and lead to minimal antihypertensive efficacy as monotherapies. Instead, they are often used with other agents to correct K^+^ deficiency^14^. Aldosterone can also affect aldosterone-sensitive apical renal transporters, namely NCC as well as ENaC^95^. Cirrhosis is accompanied with a hyperaldosteronism status that plays a vital role in the pathogenesis of sodium retention and ascites formation in cirrhosis^95^ which is consistent with our results that revealed significant hyperaldosteronism in cirrhotic rats with plasma aldosterone levels 1.9 folds higher than those observed with normal rats. Consequently, the aldosterone-sensitive ENaC renal expression was significantly elevated in untreated cirrhotic rats. Though all tested extracts, except for the lowest tested dose of At-ar, showed significant reduction in plasma aldosterone levels, the only one that significantly reduces ENaC renal expression compared to the positive control rats was the highest tested dose of At-rt. This indicates that the significant reduction in plasma aldosterone level observed in rats treated with different extracts most probably affected the binding to aldosterone receptor or the aldosterone-sensitive NCC rather than ENaC. ENaC expression was mostly decreased by the influence of eriodyctiol-7-O-glucoside, vitexin and malic acid. All of the three top scoring compounds are present in At-rt, consequently, it was the only extract capable of significantly decreasing the expression of ENaC. Unlike vitexin and malic acid, the correlation between eriodyctiol-7-O-glucoside and diuresis was not formerly studied. Pure vitexin exerted potent hypotensive effect in volunteers and rabbits through its diuretic effect^100^. In addition, extracts containing malic acid induced significant diuresis^101^. Plasma aldosterone reduction was influenced by the action of linolenic acid, rhamnocitrin-O-glucoside and calycosin. As-ar and At-rt contain two of the top active compounds against aldosterone (linolenic acid and calycosin) and resulted in lower levels of plasma aldosterone. In support with our results, linolenic acid was found to affect aldosterone level; the oxidized derivatives of linoleic acid showed biphasic action, stimulating aldosterone production at low concentrations and inhibiting it at higher concentrations^102^. Accordingly, it may be inferred that linolenic acid is present As-ar and At-rt at high concentration to cause the observed inhibitory effect on aldosterone production. While linolenic acid seems to act via direct modulation of steroid synthesis and aldosterone production by adrenal cells, rhamnocitrin-O-glucoside and calycosin mostly act through affecting renin–angiotensin–aldosterone system. Rhamnocitrin and calycosin downregulate angiotensin (Ag) II/Ag converting enzyme (ACE)/Ag II type I receptor pathway and upregulate Ag 1–7/ACE2/Mas receptor pathway with subsequent inhibition of aldosterone release from adrenal cortex^103,104^.

Induction of cirrhosis in the current model caused significant hyponatremia. Hyponatremia is a frequent complication of advanced cirrhosis correlated to an impairment in the renal capacity to eliminate solute-free water. This causes a disproportional water to sodium retention resulting in hypoosmolality which is linked pathogenically to renal aquaporins^105^. Thus, extracts that most significantly affected renal aquaporin-1, namely As-rt-D3 and At-ar-D3, showed significant reduction in serum sodium level compared to untreated rats. As-rt-D3 was the only extract that was capable to return serum sodium to its normal levels which may be attributable to its significant downregulation of aquaporin-1.

Potassium derangement is commonly detected in cirrhotic patients with both hypokalemia and hyperkalemia occurrence is possible and are linked to unfavorable prognosis^106^. The present model of cirrhosis induction was associated with significant hypokalemia which might be linked to the significantly elevated plasma aldosterone in cirrhotic rats; circulating potassium is tightly regulated by aldosterone and in high aldosterone states, surface cells contribute to active potassium secretion following an ENaC-dependent mechanism^107,108^. Extracts that showed the greatest potassium levels (At-rt and As-ar) were those with less plasma aldosterone indicating that aldosterone is one of the dominant regulators of circulating potassium in the currently applied cirrhosis animal model. It is noteworthy to say that higher doses At-rt elevated serum potassium level to an extent that significantly exceeded average values in normal rats meaning the occurrence of hyperkalemia.

The most prominent mechanism affected by each extract to exert its diuretic effect was identified by determining the % decrease in mRNA expression of renal transporters and plasma aldosterone upon treatment with the highest tested dose of each extract in comparison to the positive control group. As-rt-D3 showed the greatest % reduction in proximal tubule transporters and high % reduction in the thick ascending limb transporter, both nephron areas are responsible for the majority of Na^+^ reabsorption and their downregulation would result in urine dilution and significant diuresis. This effect was confirmed by measuring the 24 h urine volume that was significantly higher than other tested extracts (*p* < 0.05) (data not shown). As-ar-D3 more considerably affected the distal areas of the nephron more than the proximal tubule or the thick ascending limb of the loop of Henle. Since As-ar showed relatively small effect on the proximal tubule, responsible for the greatest portion of Na^+^ reabsorption, and aquaporin-1, key regulator of water reabsorption, it caused moderate diuresis. At-ar-D3 exhibited very similar effect on renal transporters as As-rt-D3 in showing greater effect on non-aldosterone sensitive transporters, resulting in the high diuretic effect observed from the collected 24 h urinary output. Compared to As-rt-D3, At-ar-D3 showed slightly less effect on NHE-1and aquaporin-1 which accounts for the observed lesser diuresis. At-rt-D3 failed to significantly decrease the expression of most renal transporters (NHE-1, aquaporin-1, NKCC2 or NCC) accounting for the weakest diuretic effect observed in rats treated with this extract.

PH is primarily caused by the increase in resistance to portal outflow, as a consequence of hepatic cirrhosis, and secondly by an increase in splanchnic blood flow. Alterations in vascular tone play a central role in the pathophysiology of PH by contributing to increased intrahepatic resistance, hyperdynamic circulation, and expansion of the collateral circulation^109^. PH can be estimated by assessing serum biomarkers, such as serum apelin^110^. Apelin is an endogenous ligand for angiotensin-like receptor 1 (APJ) that is known to have diverse physiological and pathological effects, including regulation of cardiovascular function, fluid homeostasis and so on. Serum apelin levels have shown close relationships with both intrahepatic cirrhosis and splanchnic hemodynamics^111^. In cirrhosis, the hepatic nitric oxide (NO) levels are significantly reduced, with associated elevated sinusoidal vascular resistance. On the other hand, asymmetric dimethylarginine (ADMA), the competitive endogenous inhibitor of endothelial nitric oxide synthase (eNOS) that synthetizes NO, is metabolized by dimethylarginine dimethylaminohydrolase-1 (DDAH-1) within the liver. Impaired liver function is associated with increased blood levels of ADMA and correlates with the severity of PH and decompensation^10^. Induction of cirrhosis in the present work significantly increased the circulating level of apelin, which matches early reports^112^, indicating notable PH. Hepatic DDAH-1 was significantly decreased in rats with cirrhosis, reflecting increased availability of ADMA and NO synthesis inhibition. Under normal circumstances, apelin/APJ cause a decrease in blood pressure, but, under pathological conditions that cause vascular damage, such as in cirrhosis where elevated ADMA is a risk factor for endothelial dysfunction, apelin functions as a vasopressor peptide^113^. Apelin/APJ system induces hypertension via affecting sympathetic nervous system, renin–angiotensin–aldosterone system, endothelial injury, excessive endothelin, sodium retention and vascular remodeling^113^. Since serum apelin is elevated only after vascular damage induced by cirrhosis development, reversal of cirrhosis associated fibrosis and hepatic damage would be the driving factors for decreasing serum apelin and counteracting apelin-induced PH. Therefore, extracts that more profoundly reduced fibrosis markers and restored hepatic integrity were those that more significantly reduced serum apelin. According to the OPLS model, apelin is mosly reduced by effect of quinic acid, isorhamnetin-3-O-B-D-glucoside and caffeic acid-O-hexoside. Quinic acid is the only compound from these top active compounds that is present in As-ar, the extract that showed the greatest reduction in serum apelin, which means that this compound may possess a great capability to modulate apelin. In accordance, polyphenols, such as quinic acid, showed the ability to counteract the hazardous effects of elevated apelin in obese diabetic pregnant females^114^. Generally, decreased levels of DDAH-1 result in increased ADMA levels which is linked to up-regulation of circulating renin–angiotensin–aldosterone system components^115^, sodium retention and blood pressure increase^116^. In the present work, the activity of different extracts towards DDAH-1 may be linked to their combined diuretic effect and influence on circulating aldosterone. The compounds that revealed the greatest ability to increase the level of hepatic DDAH-1 were naringenin, isorhamnetin and quercetin-3-O-rhamnoside. All of these compounds previously have proved efficacy in modulating DDAH–ADMA–NO pathway and were all exclusively detected in At-ar, therefore, At-ar-D3 showed the highest hepatic DDAH-1 level. For instance, Naringenin had been shown to stimulate eNOS and NO in endothelial cells exposed to high glucose^117^. Isorhamnetin also showed protective effects on endothelial cell line EA.hy926 and caused upregulation of eNOS expression^118^. Further, Quercetrin affected endothelial NO^⋅^ signaling by inhibiting both arginase-1 activity and expression^119^.

## Methods

### Preparation and analysis of plants extracts

#### Collection of plant materials:

*Astragalus spinosus* and *Astragalus trigonus* whole plant materials were collected in August 2020 from Borg El Arab, Alexandria, Egypt, with permission from the Agriculture Research Center, Giza, Egypt at "9 Cairo University Road, Giza District, Giza Governorate". The plant collection was accomplished in accordance with the national guidelines. The identity of the plant materials was confirmed by Prof. Dr. Salama El Dareer, Professor of Botany, Faculty of Science, Alexandria University. A voucher specimens of *A. spinosus* and *A. trigonus* (No.: A-SP-019 and A-TR-019, respectively) were deposited at the herbarium of the Department of Pharmacognosy, Faculty of Pharmacy, Alexandria University. The roots and the aerial parts of the two species were separated and left for complete dryness at room temperature.

#### Plant extracts and samples preparation

The air-dried powdered *A. spinosus* and *A. trigonus* aerial parts (100 g from each species) and roots (50 g from each species) were separately extracted with methanol, filtered and dried under reduced pressure using rotary evaporator at 45 ˚C to afford dry solid residues (3.5, 2.8, 1.5 and 1.3 g, respectively). The four samples were prepared at (1 mg/ml) concentration using HPLC-grade methanol, filtered using membrane filters (0.2 μm). The forementioned process was repeated three times for each sample to ensure reproducibility.

#### Standard solutions for UPLC-MS quantification

The stock standard solutions of caffeic acid, malic acid, sucrose, quercetin, astragaloside I and linoleic acid were prepared as follow: an accurate weight of each compound (10 mg) was separately placed in 10-mL volumetric flask. HPLC-grade methanol was added, and the solution was diluted to the working concentrations ranging from 0.0125 to 0.625 mg mL^−1^ using the same solvent as shown in Table S2. Five μL aliquots of each standard solution were applied onto the chromatographic column and the injections were carried out in triplicates for each concentration level. The calibration curves were generated by plotting peak areas of the standards versus their concentrations.

#### High-resolution ultra-performance liquid chromatography-mass spectrometry analysis (UPLC-ESI- TQD -MS)

Separation and characterization of *Astragalus* secondary metabolites was employed using UPLC XEVO TQD instrument (Waters Corporation, Milford, MA01757 U.S.A.).

##### Chromatographic parameters and conditions

The UPLC system consists of a Waters Acquity QSM pump, a LC-2040 (Waters) autosampler, degasser and Waters Acquity CM detector. 10 μl of each of the previously prepared samples (full loop injection volume) were separately injected into the chromatographic column. Chromatographic separation was conducted using a Waters Acquity UPLC BEH C18 column (50 mm × 2.1 mm ID × 1.7 μm particle size) operating at a flow rate of 0.2 ml/min and thermostating at 30 °C.

The analyses were performed using a binary mobile phase consisted of ultrapure water + 0.1% (v/v) formic acid (Phase A) and methanol + 0.1% (v/v) formic acid (Phase B). The gradient elution programmed as follows: 0.0–2.0 min, 10% B; 2.0–5.0 min, 30% B; 5.0–15.0 min, 70% B; 22.0 min, 90% B; 22.0–25.0 min, 90% B; 26.0 min, 100% B; 26.0–29.0 min, 100% B; 30.0 min, 10% B; followed by 4 min of re-equilibration.

##### ESI–MS parameters and conditions

Electrospray ionization (ESI) source in conjunction with triple quadrupole (TQD) mass spectrometer were utilized to analyze the samples in a negative ionization mode. ESI operating conditions briefly were: capillary voltage of 3 kV, cone voltage; 35 V, the ion source temperature was 150^◦^C, the nebulizer (nitrogen gas) pressure was 35 psi, drying and sheath gas (N_2_) temperature was 440 °C and 350 °C, respectively. The drying and sheath gas flows were applied at 900 L/h and 50 L/h, respectively. The analytical run time was extended to 30 min. MS spectra were achieved by full range acquisition covering 100–1000 m/z. For automatic MS/MS fragmentation analyses of the precursor ions which were mass-selected by the first quadrupole (Q1), the collision-induced dissociation (CID) energy was ramped from 30 to 70 eV using nitrogen gas as a collision gas in the second quadrupole collisional cell (Q2). Finally, the daughter ions yielded from CID are consequently related to the molecular structure of the precursor ions and can be monitored by a third quadrupole mass analyzer (Q3).

##### Characterization of compounds

For annotation of secondary metabolites of *A. spinosus* and *A. trigonus* aerial parts and roots samples, comparison of the quasi-molecular ions and MS/MS data together with retention times with reference literature was implemented.

### Mass spectrometry data processing for multivariate data analysis

MZmine 2.0 (http://mzmine.sourceforge.net/) freely downloadable data analysis software was utilized for relative quantitation of the UPLC-MS derived metabolic fingerprints of the four *Astragalus* extracts as well as rapid processing of the raw data according to the protocol adopted by Ghallab et al.^120^.

#### Chemometric analysis of metabolic fingerprints

The UPLC-MS processed data were subjected to multivariate data analyses for complex data mining and dimensionality reduction of *Astragalus* extracts metabolic fingerprints. The data matrix was imported to SIMCA-P software (Version 14.0, Umetrics, Umea, Sweden) for building various chemometric models.

##### Unsupervised pattern recognition analysis

Principal Component Analysis (PCA) and Hierarchial Clustering Analysis (HCA) were used as exploratory data approaches, where PCA summarized the information in the data set through searching the direction of maximum variance through using a small number of orthogonal latent variables. PCA was performed using nine components and 0.95 confidence interval for Q and T2 hotelling limits for outliers. HCA-heat map based on average-linkage method was used for building dendrograms with the Euclidean distance and the centered correlation metric using MetaboAnalyst (https://www.metaboanalyst.ca/).

### *In-vivo* experiments

#### Materials

Carbon tetrachloride (CCl_4_) 98% was from Iso-Chem fine chemicals (Vert-le-Petit, France). Albumin colorimetric kit was purchased from BioMed (Hannover, Germany). Alanine aminotransferase (ALT) and aspartate aminotransferase (AST) colorimetric kits were from Biosystems (Barcelona, Spain). Rat apelin and rat dimethylarginine dimethylaminohydrolase 1 (DDAH1) ELISA kits were purchased from Cusabio (Houston, TX 77,054, USA). ELISA kits for transforming growth factor beta (TGF-ꞵ) and retinoid-X receptor (RXR) were from Mybiosource (San Diego, California, United States), while that for matrix metalloproteinase 2 (MMP-2) was from Elabscience (Houston, Texas, United States). Colorimetric assay kits of sodium and potassium were supplied from Linear chemicals (Barcelona, Spain). Trizole plus RNA purification kit was from Invitrogen—ThermoFisher (Carlsbad, United States). High-capacity cDNA reverse transcription kit, SYBR green PCR master mix and SYBR green RT-PCR reagents kits were from Qiagen (Hilden, Germany).

#### Animals

The current study was performed on 112 healthy male Sprague Dawley rats of a local bred strain, weighing 200 ± 20 g each. The rats were purchased and housed in the animal house of Faculty of Pharmacy, Pharos University in Alexandria (PUA); (Alexandria, Egypt), under standard environmental conditions of light and temperature. Rats were allowed to acclimatize for a period of one week prior to experimentation. The use of animals and all experimental procedures were approved by the PUA Research Ethics Committee and were performed in strict accordance with the regulations of ARRIVE guidelines. All methods were performed in accordance with ARRIVE guidelines and regulations.

#### Experimental design

Animals were divided into 14 groups; each composed of eight rats, as follows: Plain normal control group (eight rats) that received 1 mL saline i.p. daily. Liver cirrhosis was induced in the remaining rats by intraperitoneal administration of CCl_4_ after dilution with mineral oil at the ratio 1:6 as follows: The first 10 doses were received every 5 days, the subsequent 10 doses were administered every 4 days, and the latest 7 doses were given every 3 days^121^. Different doses (40, 80 and 160 mg/kg) of *A. spinosus* and *A. trigonus* aerial parts and roots extracts were orally administered starting from the 10th week to the end of the experiment (16th week).

At the end of experimentation period, all rats were put in metabolic cages for 24‐hr urine collection and then euthanized under desflurane anesthesia. Blood samples were collected for sera and plasma separation then killed by decapitation. Livers were quickly isolated and an appropriate part was sectioned and kept in 10% formal-saline for Masson’s trichrome staining and histopathological examination for confirming cirrhosis induction defined by formation of hepatic nodules. The remaining parts of the livers and kidneys were stored in liquid nitrogen and then in −80 °C until performing quantitative RT-PCR (qRT-PCR), ELISA and biochemical testing.

#### Effect of Different *Astragalus* Extracts on Liver Cirrhosis Markers

##### Determination of hepatic TGF-ꞵ, MMP-2 and RXR-α by ELISA

TGF-ꞵ, MMP-2 and RXR-α levels were assessed using ELISA kits following manufacturers’ guidance. Levels were assayed in the supernatant obtained following centrifugation of the homogenized liver tissue (20% homogenates in PBS (pH7.4)) in cooling-centrifuge (Centurion-scientific-K3series, UK) at 15,000 rpm at 5 °C for 5 min.

##### Biochemical assessment of serum ALT, AST and albumin

ALT, AST concentrations (U/L) and albumin (g/dL) in the sera obtained from different groups were assayed colorimetrically using colorimetric assay kit according to instructions provided by suppliers.

#### Prediction of the diuresis mechanism of different *Astragalus* extracts

##### Assessment of mRNA expression (2^-ΔΔCt) of different renal transporters acting at different nephron parts using qRT-PCR

Using real time one step qRT-PCR, the renal expression of Aquaporin 1, Na–K-Cl co-transporter 2 (NKCC 2), sodium-chloride symporter (NCC), sodium–hydrogen antiporter 1 (NHE1) and epithelial sodium channel (ENaC) was assessed. Total RNA isolation using total RNA isolation kit and purity determination at 260 and 280 nm were performed as previously reported^122^. Reverse transcription was done using High-capacity cDNA reverse transcription kit (Applied Biosystems; California, USA). Then qRT-PCR was performed, using SYBR Green PCR master mix and SYBR green RT-PCR reagents kit according to manufacturer’s instructions, to amplify the cDNA using sets of specific primer for each gene (Table 3). During the extension step, data acquisition was collected using Rotor-Gene Q-Pure detection, software version 2.1.0 (build 9); (Qiagen; Hilden, Germany). Quantification was expressed as normalized ratio of the relative expression of target genes relative to reference gene in the same sample by calculating the threshold cycles (Ct) values using ΔΔCt method_ENREF_41.

**Table 3:** Used oligonucleotide primers in the present study for RT-PCR analysis.

Primer		Sequence	Product length	Accession number
aquaporin 1 (Aqp1)	Forward	5’-TTGCAAGGACCTGATGCTGT-3’	139	NM_012778.1
	Reverse	5’-TAACGGCACAGTGGTAGAGC-3’		
NKCC2 (Slc12a1)	Forward	5’-AAGCGGGAATTGGTCTTGGA-3’	81	NM_001270617.1
	Reverse	5’-TTGCAGAAGTTGACAACCCAGT-3’		
NCC (Slc12a3)	Forward	5'- TCCCAGTATTGGGTGTGCAA-3'	115	NM_019345.3
	Reverse	5'- CACATGGGTCCTCAGGATGG-3'		
NHE1 (Slc9a1)	Forward	5'-ACCCCTCGTCTAGACCACTC-3'	132	NM_012652.1
	Reverse	5'-CTCAGGGGTTGGACAGACAC-3'		
β-actin	Forward	5'- ATCATTGCTCCTCCTGAGCG-3'	179	NM_031144.3
	Reverse	5'- GAAAGGGTGTAAAACGCAGCTC-3'		

##### Determination of plasma Aldosterone

Plasma aldosterone (nM) obtained from different groups was assayed using ELISA kit according to instructions provided by supplier.

##### Determination of serum electrolytes

Serum sodium (mM) and potassium (mM) were analyzed spectrophotometrically by using colorimetric assay kits following manufacturer’s instructions.

#### Effect of Different *Astragalus* Extracts on Portal Hypertension Markers (serum apelin and hepatic DDAH1 by ELISA)

Both apelin (ng/mL) and DDAH1 (pg/g protein) were assessed in supernatants, separated by centrifuging 20% liver homogenates from different groups, respectively, using ELISA kits following steps provided by their suppliers.

#### Statistical analysis of the data

Data were analyzed using IBM SPSS software package version 20.0*.* (Armonk, NY: IBM Corp). The normality of distribution was verified using Kolmogorov–Smirnov test. Quantitative data were described using mean and SD. Significance of the obtained results was judged at the 5% level. In order to compare between more than two groups, F-test (ANOVA) was used for normally distributed quantitative variables, followed by Post Hoc test. Outliers were excluded from data set.

### Supervised pattern recognition analysis

Orthogonal projection to latent structures (OPLS) was utilized to model the metabolite differences among the different *Astragalus* extracts and to investigate chemical variability within the X-matrix (via employing LC–MS data as independent variables) that were related to the investigated pharmacological activities (Y- dependent variables). Accordingly, the algorithm exposed the main efficacy associated markers via biplots and coefficient plots.

## Conclusion

Different extracts of *A. spinosus* and *A. trigonus* roots and aerial parts significantly ameliorate cirrhosis/fibrosis and associated PH to variable extents. They also induced powerful diuresis by inconsistent mechanisms that affect different nephron portions. This study represents the first approach to reveal the metabolites variation among *Astragalus* species using UPLC-ESI- TQD -MS coupled with multivariate data analysis. The study showed that the differences among studied accessions mainly emanate from saponins and flavonoid content. Based on MS2 analysis, a number of phenolic acids, saponins, flavonoids and fatty acids were reported for the first time in *A. spinosus* and *A. trigonus*. OPLS models assisted in identifying the exact metabolites from each extract responsible for each pharmacological action.

## Supplementary Information

Supplementary Information 1.

Supplementary Information 2.

Supplementary Information 3.

## Data Availability

All data generated or analyzed during this study are included in this published article and its supplementary information files. The datasets (RNA sequences) analysed during the current study are available in the GenBank repository, https://www.ncbi.nlm.nih.gov/genbank/, accession numbers are included in Table 3.

## Abbreviations

ADMA: Asymmetric dimethylarginine
DDAH-1: Dimethylarginine dimethylaminohydrolase-1
ECM: Extracellular matrix
eNOS: Endothelial nitric oxide synthase
ENaC: Epithelial sodium channel
HCA: Hierarchial clustering analysis
HSCs: Hepatic stellate cells
MMPs: Matrix metalloproteinases
NCC: sodium-chloride symporter
NHE1: Sodium–hydrogen antiporter 1
NKCC 2: Na–K–Cl co-transporter 2
NO: Nitric oxide
OPLS: Orthogonal projection to latent structures
PCA: Principal component analysis
PH: Portal hypertension
RXRs: Retinoid X receptors
TGF-β: Transforming growth factor-β
