# Supplementary material for: Alleviation of liver cirrhosis and associated portal-hypertension by Astragalus species in relation to their UPLC-MS/MS metabolic profiles: a mechanistic study
Source: Sci Rep. 2022 Jul 13;12:11884. doi: 10.1038/s41598-022-15958-1 (PMC9279505; doi:10.1038/s41598-022-15958-1)
Supplement: Supplementary file 1 — Supplementary Information 1. [file 41598_2022_15958_MOESM1_ESM.docx]

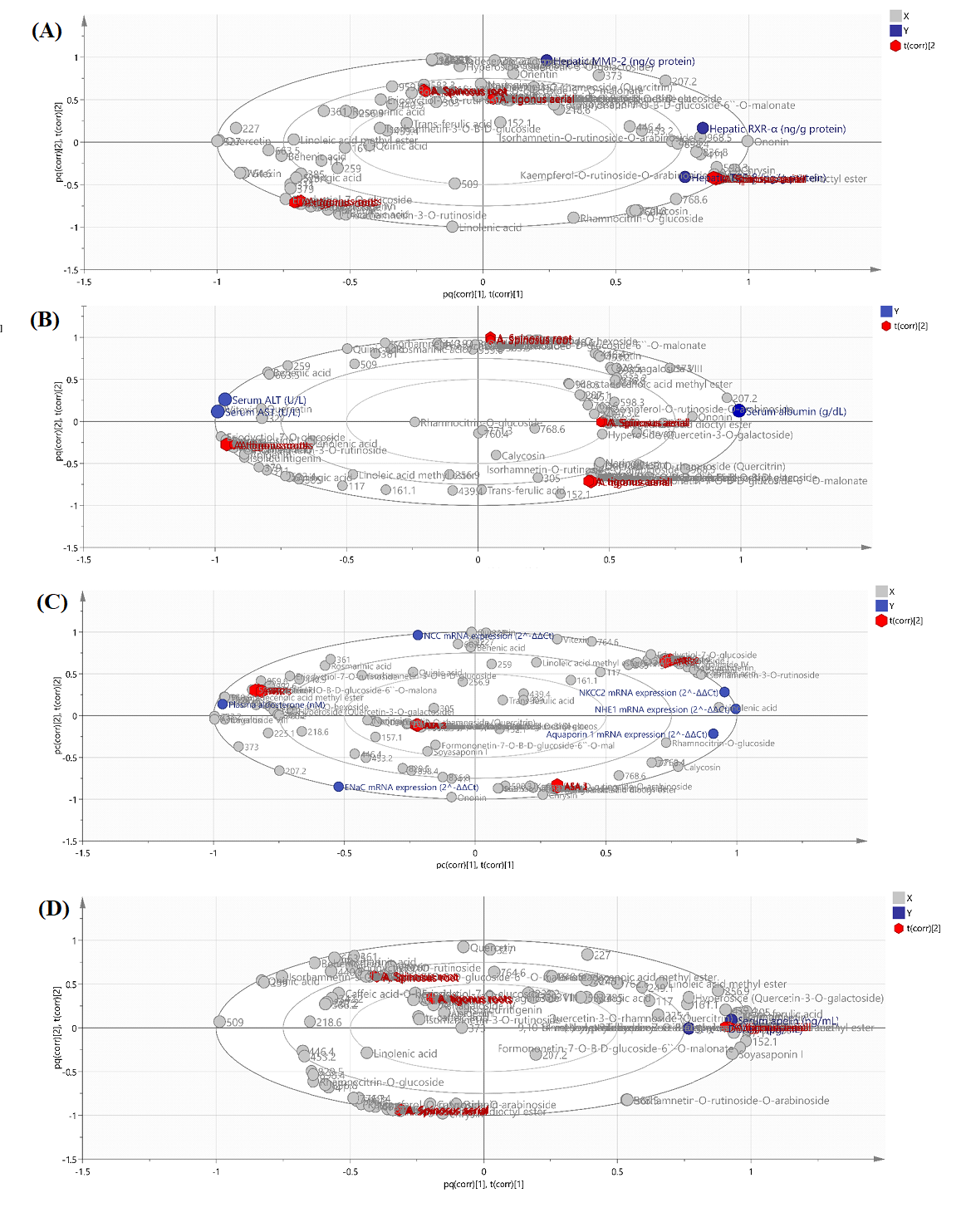
**Supplementary fig.1: Biplot of all performed OPLS models of different *Astragalus* extracts**

A: liver cirrhosis markers, B: serum markers of hepatocytes integrity and liver synthetic function, C: Renal transporters, D: portal hypertension markers

The figure was created using SIMCA-P software (Version 14.0, Umetrics, Umea, Sweden). (<https://www.bioz.com/result/simca%20p%20software%20version%2014%200/product/Umetrics>)


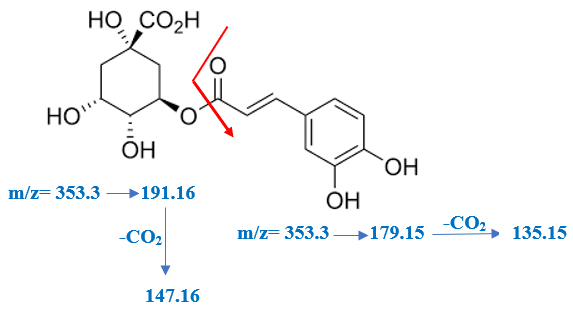


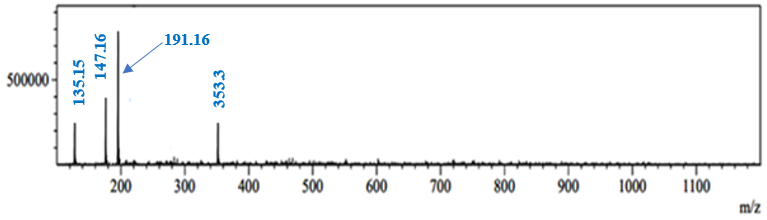


**Supplementary fig.2: MS/MS spectrum of chlorogenic acid and its supposed fragmentation pattern**

MS/MS spectrum was obtained from Waters Acquity CM detector. The structure of chlorogenic acid was drawn using ChemDraw software (https://perkinelmerinformatics.com/products/research/chemdraw). The illustrated fragmentation pattern was recognized by comparison of the quasi-molecular ions and MS/MS data with reference literature.


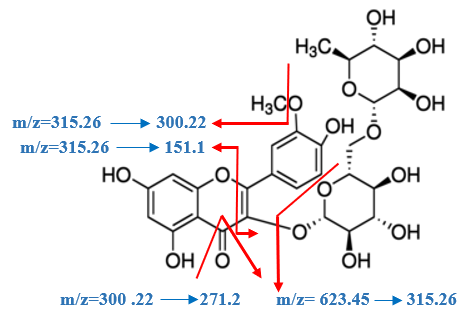


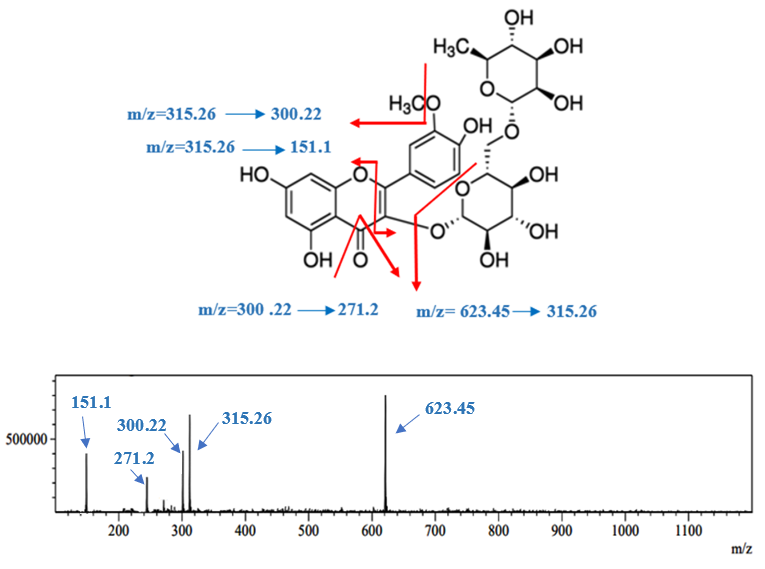


**Supplementary fig.3: MS/MS spectrum of** **Isorhamnetin-3-O-rutinoside and its supposed fragmentation pattern**

MS/MS spectrum was obtained from Waters Acquity CM detector. The structure of Isorhamnetin-3-O-rutinoside was drawn using ChemDraw software (https://perkinelmerinformatics.com/products/research/chemdraw). The illustrated fragmentation pattern was recognized by comparison of the quasi-molecular ions and MS/MS data with reference literature.
