# Supplementary material for: Alleviation of liver cirrhosis and associated portal-hypertension by Astragalus species in relation to their UPLC-MS/MS metabolic profiles: a mechanistic study
Source: Sci Rep. 2022 Jul 13;12:11884. doi: 10.1038/s41598-022-15958-1 (PMC9279505; doi:10.1038/s41598-022-15958-1)
Supplement: Supplementary file 3 — Supplementary Information 3. [file 41598_2022_15958_MOESM3_ESM.docx]

**Table S2: Linearity and sensitivity parameters for caffeic acid, malic acid, sucrose, quercetin, astragaloside I and linoleic acid used as *Astragalus* standards**

| **Compound** | **Linearity range (mg.mL^-1^)** | **Slope (b)** | **Intercept (a)** | **r** | **LOD (mg mL^-1^)** | **LOQ (mg mL^-1^)** |
| --- | --- | --- | --- | --- | --- | --- |
| **Caffeic acid** | **0.0125-0.25** | **32*10^6^** | **35** | **0.996** | **0.005** | **0.0125** |
| **Malic acid** | **0.025-0.536** | **2.87*10^8^** | **– 8.35*10^5^** | **0.993** | **0.010** | **0.025** |
| **Sucrose** | **0.0325-0.625** | **41.71*10^6^** | **103907** | **0.997** | **0.009** | **0.0325** |
| **Quercetin** | **0.02-0.525** | **20.51*10^6^** | **-107** | **0.995** | **0.012** | **0.02** |
| **Astragaloside I** | **0.055-0.625** | **5170** | **815** | **0.994** | **0.015** | **0.055** |
| **Linoleic acid** | **0.0135-0.255** | **30.71*10^5^** | **1499** | **0.995** | **0.003** | **0.0135** |

Experimental conditions as in Section 4.1.3. For each calibration curve (except astragaloside I) the equation is y = ax + b, where y is the peak area, x the concentration of the standard (mg.mL^-1^), a is the intercept, b is the slope and r is the correlation coefﬁcient.

For astragaloside I, the regression equation is the same as above except that y and x refer to the logarithmic values of the peak area and concentration of the standard, respectively.

**Table S3: Content of identified compounds in *Astragalus* samples analyzed by UPLC-MS (data are expressed as mg.mL^-1^)**

| **Metabolites** | **ASA** | **ASR** | **ATA** | **ATR** |
| --- | --- | --- | --- | --- |
| **Caffeic acid-O-hexoside** | 0 | 0 | 0 | ˂ LOD |
| **Quinic acid** | 0.069761406 | **0** | 0.121269844 | 0.005666938 |
| **Malic acid** | **0** | **0** | 0.012191003 | **0** |
| **Gallic acid** | **0** | 0.031796906 | **0** | **0** |
| **Chlorogenic acid** | **0** | **0** | **0** | 0.053670344 |
| **tr-caffeic acid** | 0.040453781 | **0** | 0.167039875 | 0.013108219 |
| **Syringic acid** | **0** | **0** | 0.077262563 | 0.045614094 |
| **Rosmarinic acid** | **0** | 0.156604844 | 0.058979281 | **0** |
| **Kaempferol-O-rutinoside-O-arabinoside** | 0.080817527 | 0.048073086 | 0.042111945 | 0.040019113 |
| **Isorhamnetin-O-rutinoside-O-arabinoside** | 0.030471672 | **0** | **0** | 0.028970258 |
| **Dihexose** | **0** | **0** | **0** | 0.011421793 |
| **Rutin (quercetin-3-O-rutinoside)** | **0** | **0** | 0.851673476 | 0.041195563 |
| **Eriodyctiol-7-O-rutinoside** | **0** | 0.721675963 | 0.146471234 | ˂ LOD |
| **Isorhamnetin-3-O-rutinoside** | 0.187020283 | ˂ LOD | 0.685290054 | 0.06491648 |
| **ferulic acid** | **0** | **0** | 0.027475406 | 0.062650844 |
| **Calycosin-7-O-B-D-glucoside-6``-O-malonate** | **0** | 0.054511312 | **0** | **0** |
| **Formononetin-7-O-glucoside-6``-O-malonate** | 0.008002243 | **0** | **0** | 0.033162994 |
| **Naringin** | **0** | 0.030476499 | **0** | 0.142951195 |
| **Hyperoside (Quercetin-3-O-galactoside)** | **0** | **0** | **0** | ˂ LOD |
| **Orientin (Luteolin-8-C-glucoside)** | 0.020534764 | 0.055137543 | **0** | 0.023393662 |
| **Astragalin (Kaempferol-3-O-glucoside)** | **0** | **0** | **0** | 0.017793272 |
| **Eriodyctiol-7-O-glucoside** | **0** | 0.021659239 | 0.245382301 | **0** |
| **Isorhamnetin-3-O-B-D-glucoside** | **0** | 0.188186982 | 0.116126329 | 0.044069576 |
| **Quercetin-3-O-rhamnoside (Quercitrin)** | **0** | **0** | **0** | 0.019173184 |
| **Vitexin (Apigenin-8-C-glucoside)** | **0** | 0.074799317 | 0.146365822 | 0.017915651 |
| **Rhamnocitrin-O-glucoside** | 0.144247196 | 0.042514432 | 0.111198147 | 0.015473038 |
| **Pratensein-7-O-B-D-glucoside** | **0** | **0** | **0** | 0.149732667 |
| **Ononin (Formononetin-7-O-glucoside)** | 0.184125939 | 0.066083325 | **0** | 0.07674744 |
| **9,10-dimethoxypterocarpan-3-O-B-D-glucoside** | **0** | **0** | **0** | 0.021441589 |
| **(-)-methylinissolin-3-O-B-D-glucoside** | **0** | **0** | **0** | 0.019018479 |
| **Myricetin** | 0.040338957 | 0.037400098 | 0.389034227 | 0.038063823 |
| **Quercetin** | **0** | 0.073502145 | 0.09521843 | 0.04649922 |
| **Silymarin** | **0** | **0** | **0** | 0.03864588 |
| **Naringenin** | **0** | **0** | **0** | 0.108627109 |
| **Hesperetin** | **0** | **0** | **0** | ˂ LOD |
| **Daidzein** | **0** | **0** | **0** | 0.044447196 |
| **Isoliquiritigenin** | 0.027072062 | **0** | 0.163721599 | 0.033551682 |
| **Kaempferol** | **0** | 0.267383569 | **0** | **0** |
| **Apigenin** | 0.038648854 | **0** | 0.197404632 | 0.028607021 |
| **Isorhamnetin** | **0** | **0** | **0** | 0.022932033 |
| **Chrysin** | 0.093231887 | **0** | **0** | 0.019551048 |
| **Calycosin** | 0.142197806 | **0** | 0.081254169 | 0.041725597 |
| **Astragaloside IV** | 0.69730418 | **0** | 0.697617995 | 0.697250379 |
| **Astragaloside II** | 0.697731111 | **0** | **0** | 0.697228744 |
| **Soyasaponin I** | 0.697345333 | 0.697337082 | 0.697563048 | 0.697302683 |
| **Astragaloside I** | 0.697258552 | 0.69741317 | **0** | 0.697337974 |
| **Astragaloside VIII** | 0.697410696 | 0.697184816 | **0** | 0.697558561 |
| **Linolenic acid** | 0.156093455 | **0** | 0.235443829 | **0** |
| **9,10,13-trihydroxy-11-octadecenoic acid** | **0** | **0** | **0** | 0.028182025 |
| **Palmitic acid methyl ester** | **0** | **0** | **0** | 0.035291436 |
| **14-methyl pentadecanoic acid methyl ester** | **0** | **0** | **0** | 0.305468251 |
| **Linoleic acid** | **0** | 0.384038098 | **0** | **0** |
| **Linoleic acid methyl ester** | 0.340525562 | 0.724313904 | 1.56379746 | 1.619335721 |
| **16-octadecenoic acid methyl ester** | **0** | 0.037369261 | **0** | 0.025807555 |
| **Hexanedioic acid dioctyl ester** | 2.62570661 | **0** | **0** | 0.078888961 |
| **Behenic acid** | 0.180512862 | 0.732772061 | 0.760663953 | 0.099844676 |

**Table S4: Comparison between the different studied groups according to Serum sodium (mM)**

|  | **Normal** | **Cirr+no treatment** | **Cirr+**  **As-rt-**  **D1** | **Cirr+**  **As-rt-**  **D2** | **Cirr+**  **As-rt-**  **D3** | **Cirr+**  **As-ar-D1** | **Cirr+**  **As-ar-D2** | **Cirr+**  **As-ar-D3** | **Cirr+**  **AT-rt-D1** | **Cirr+**  **AT-rt-D2** | **Cirr+**  **AT-rt-D3** | **Cirr+**  **AT-ar-D1** | **Cirr+**  **AT-ar-D2** | **Cirr+**  **AT-ar-D3** |
| --- | --- | --- | --- | --- | --- | --- | --- | --- | --- | --- | --- | --- | --- | --- |
| Mean | 152.34 | 145.73 | 147.51 | 150.13 | 151.98 | 146.01 | 146.08 | 146.16 | 145.81 | 145.88 | 145.96 | 146.08 | 147.36 | 149.01 |
| ±SD. | 1.64 | 2.07 | 1.19 | 0.82 | 1.29 | 0.57 | 0.61 | 0.93 | 0.57 | 0.61 | 0.93 | 0.61 | 0.92 | 1.19 |
| **F (p)** | **37.432^*^ (<0.001^*^)** | | | | | | | | | | | | | |
| **p_1_** |  | <0.001^*^ | <0.001^*^ | 0.007^*^ | 1.000 | <0.001^*^ | <0.001^*^ | <0.001^*^ | <0.001^*^ | <0.001^*^ | <0.001^*^ | <0.001^*^ | <0.001^*^ | <0.001^*^ |
| **p_2_** |  |  | 0.075 | <0.001^*^ | <0.001^*^ | 1.000 | 1.000 | 1.000 | 1.000 | 1.000 | 1.000 | 1.000 | 0.150 | <0.001^*^ |
| **p_3_** |  |  |  | <0.001^*^ | <0.001^*^ | 0.260 | 0.324 | 0.427 | 0.113 | 0.150 | 0.215 | 0.324 | 1.000 | 0.260 |
| **p_4_** |  |  |  |  | 0.054 | <0.001^*^ | <0.001^*^ | <0.001^*^ | <0.001^*^ | <0.001^*^ | <0.001^*^ | <0.001^*^ | <0.001^*^ | 0.732 |
| **p_5_** |  |  |  |  |  | <0.001^*^ | <0.001^*^ | <0.001^*^ | <0.001^*^ | <0.001^*^ | <0.001^*^ | <0.001^*^ | <0.001^*^ | <0.001^*^ |
| **p_6_** |  |  |  |  |  |  | 1.000 | 1.000 | 1.000 | 1.000 | 1.000 | 1.000 | 0.427 | <0.001^*^ |
| **p_7_** |  |  |  |  |  |  |  | 1.000 | 1.000 | 1.000 | 1.000 | 1.000 | 0.507 | <0.001^*^ |
| **p_8_** |  |  |  |  |  |  |  |  | 1.000 | 1.000 | 1.000 | 1.000 | 0.622 | <0.001^*^ |
| **p_9_** |  |  |  |  |  |  |  |  |  | 1.000 | 1.000 | 1.000 | 0.215 | <0.001^*^ |
| **p_10_** |  |  |  |  |  |  |  |  |  |  | 1.000 | 1.000 | 0.272 | <0.001^*^ |
| **p_11_** |  |  |  |  |  |  |  |  |  |  |  | 1.000 | 0.366 | <0.001^*^ |
| **p_12_** |  |  |  |  |  |  |  |  |  |  |  |  | 0.507 | <0.001^*^ |
| **p_13_** |  |  |  |  |  |  |  |  |  |  |  |  |  | 0.142 |

ANOVA test was used to compare between the different groups with Post Hoc Test (Tukey) to compare different groups. F: F for ANOVA test *: Statistically significant at p≤0.05, n=8. (Cirr.: induced cirrhosis by i.p. administration of CCl_4_ (diluted 1:6 with mineral oil) as follows: The first 10 doses were received every 5 days, the subsequent 10 doses were administered every 4 days, and the last 7 doses were given every 3 days, As: *Astragalus spinosus*, AT: *Astragalus trigonus*, ar: aerial organs, rt: root organs, D1: low dose, D2: medium dose, D3: high dose, p_1_: p value for comparing between N and each other groups, p_2_: p value for comparing between C and each other groups, p_3_: p value for comparing between As-rt-D1 and each other groups, p_4_: p value for comparing between As-rt-D2 and each other groups, p_5_: p value for comparing between As-rt-D3 and each other groups, p_6_: p value for comparing between As-ar-D1 and each other groups, p_7_: p value for comparing between As-ar-D2 and each other groups, p_8_: p value for comparing between As-ar-D3 and each other groups, p_9_: p value for comparing between AT-rt-D1 and each other groups, p_10_: p value for comparing between AT-rt-D2 and each other groups, p_11_: p value for comparing between AT-rt-D3 and each other groups, p_12_: p value for comparing between AT-ar-D1 and each other groups, p_13_: p value for comparing between AT-ar-D2 and AT-ar-D3.

**Table S5: Comparison between the different studied groups according to Serum potassium (mM)**

|  | **Normal** | **Cirr+no treatment** | **Cirr+**  **As-rt-**  **D1** | **Cirr+**  **As-rt-**  **D2** | **Cirr+**  **As-rt-**  **D3** | **Cirr+**  **As-ar-D1** | **Cirr+**  **As-ar-D2** | **Cirr+**  **As-ar-D3** | **Cirr+**  **AT-rt-D1** | **Cirr+**  **AT-rt-D2** | **Cirr+**  **AT-rt-D3** | **Cirr+**  **AT-ar-D1** | **Cirr+**  **AT-ar-D2** | **Cirr+**  **AT-ar-D3** |
| --- | --- | --- | --- | --- | --- | --- | --- | --- | --- | --- | --- | --- | --- | --- |
| Mean | 6.33 | 5.39 | 5.96 | 6.19 | 6.31 | 5.76 | 6.23 | 6.34 | 5.89 | 6.91 | 7.29 | 5.49 | 5.75 | 5.91 |
| ±SD. | 0.49 | 0.25 | 0.21 | 0.25 | 0.40 | 0.21 | 0.23 | 0.34 | 0.25 | 0.40 | 0.25 | 0.25 | 0.21 | 0.40 |
| **F (p)** | **22.088^*^ (<0.001^*^)** | | | | | | | | | | | | | |
| **p_1_** |  | <0.001^*^ | 0.533 | 1.000 | 1.000 | 0.029^*^ | 1.000 | 1.000 | 0.232 | 0.018^*^ | <0.001^*^ | <0.001^*^ | 0.023^*^ | 0.319 |
| **p_2_** |  |  | 0.023^*^ | <0.001^*^ | <0.001^*^ | 0.476 | <0.001^*^ | <0.001^*^ | 0.090 | <0.001^*^ | <0.001^*^ | 1.000 | 0.533 | 0.058 |
| **p_3_** |  |  |  | 0.973 | 0.591 | 0.990 | 0.914 | 0.476 | 1.000 | <0.001^*^ | <0.001^*^ | 0.135 | 0.983 | 1.000 |
| **p_4_** |  |  |  |  | 1.000 | 0.273 | 1.000 | 0.999 | <0.803^*^ | 0.001^**^ | <0.001^*^ | 0.001^*^ | 0.232 | 0.883 |
| **p_5_** |  |  |  |  |  | 0.037^**^ | 1.000 | 1.000^*^ | <0.273^*^ | 0.014^*^ | <0.001^*^ | <0.001^*^ | 0.029^*^ | 0.368 |
| **p_6_** |  |  |  |  |  |  | <0.001^*^ | <0.001^*^ | 1.000 | <0.001^*^ | <0.001^*^ | <0.001^*^ | <0.001^*^ | <0.001^*^ |
| **p_7_** |  |  |  |  |  |  |  | 1.000 | 0.648 | 0.002^*^ | 0.163 | 0.001^*^ | 0.135 | 0.755 |
| **p_8_** |  |  |  |  |  |  |  |  | 0.196 | 0.023^*^ | 0.023^*^ | <0.001^*^ | 0.018^*^ | 0.273 |
| **p_9_** |  |  |  |  |  |  |  |  |  | <0.001^*^ | <0.001^*^ | 0.368 | 1.000 | 1.000 |
| **p_10_** |  |  |  |  |  |  |  |  |  |  | 0.476 | <0.001^*^ | <0.001^*^ | <0.001^*^ |
| **p_11_** |  |  |  |  |  |  |  |  |  |  |  | 0.883 | 1.000 | 0.999 |
| **p_12_** |  |  |  |  |  |  |  |  |  |  |  |  | 0.914 | 0.273 |
| **p_13_** |  |  |  |  |  |  |  |  |  |  |  |  |  | 0.999 |

ANOVA test was used to compare between the different groups with Post Hoc Test (Tukey) to compare different groups. F: F for ANOVA test *: Statistically significant at p≤0.05, n=8. (Cirr.: induced cirrhosis by i.p. administration of CCl_4_ (diluted 1:6 with mineral oil) as follows: The first 10 doses were received every 5 days, the subsequent 10 doses were administered every 4 days, and the last 7 doses were given every 3 days, As: *Astragalus spinosus*, AT: *Astragalus trigonus*, ar: aerial organs, rt: root organs, D1: low dose, D2: medium dose, D3: high dose, p_1_: p value for comparing between N and each other groups, p_2_: p value for comparing between C and each other groups, p_3_: p value for comparing between As-rt-D1 and each other groups, p_4_: p value for comparing between As-rt-D2 and each other groups, p_5_: p value for comparing between As-rt-D3 and each other groups, p_6_: p value for comparing between As-ar-D1 and each other groups, p_7_: p value for comparing between As-ar-D2 and each other groups, p_8_: p value for comparing between As-ar-D3 and each other groups, p_9_: p value for comparing between AT-rt-D1 and each other groups, p_10_: p value for comparing between AT-rt-D2 and each other groups, p_11_: p value for comparing between AT-rt-D3 and each other groups, p_12_: p value for comparing between AT-ar-D1 and each other groups, p_13_: p value for comparing between AT-ar-D2 and AT-ar-D3.
